# Optimized Spatial Configuration of Heterogeneous Biocatalysts Maximizes Cell-Free Biosynthesis of ω-Hydroxy and ω-Amino Acids

**DOI:** 10.1021/acssuschemeng.4c02396

**Published:** 2024-06-10

**Authors:** Javier Santiago-Arcos, Susana Velasco-Lozano, Eleftheria Diamanti, Ana I. Benítez-Mateos, Daniel Grajales-Hernández, Francesca Paradisi, Fernando López-Gallego

**Affiliations:** † Heterogeneous Biocatalysis Group, CIC biomaGUNE, Edificio Empresarial “C”, Paseo de Miramón 182, 20009 Donostia, Spain; ‡ Instituto de Síntesis Química y Catálisis Homogénea (ISQCH), CSIC-Universidad de Zaragoza, C/Pedro Cerbuna, 12, 50009 Zaragoza, Spain; § Department of Chemistry, Biochemistry and Pharmaceutical Sciences, University of Bern, Freiestrasse 3, 3012 Bern, Switzerland; ∥ Aragonese Foundation for Research and Development (ARAID), 50018 Zaragoza, Spain; ⊥ IKERBASQUE, Basque Foundation for Science, 48009 Bilbao, Spain

**Keywords:** enzyme immobilization, flow biocatalysis, alcohol dehydrogenases, multienzyme cascades, cofactor recycling, hydrogen peroxide

## Abstract

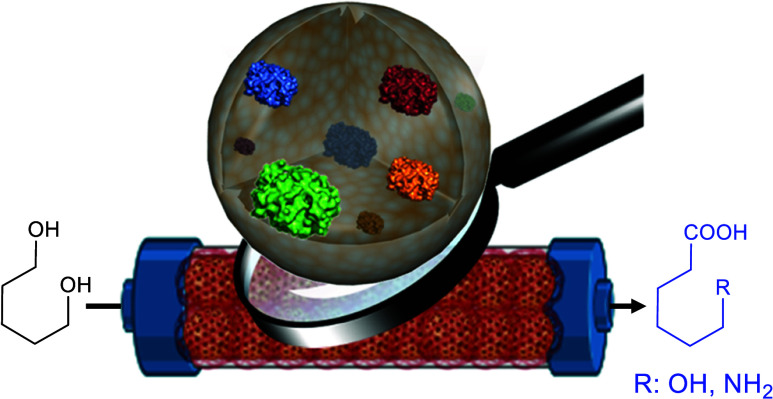

Cell-free biocatalysis is gaining momentum in producing value-added chemicals, particularly in stepwise reaction cascades. However, the stability of enzyme cascades in industrial settings is often compromised when free enzymes are involved. In this study, we have developed a stable multifunctional heterogeneous biocatalyst coimmobilizing five enzymes on microparticles to transform 1,ω-diols into 1,ω-hydroxy acids. We improved the operational efficiency and stability of the heterogeneous biocatalyst by fine-tuning the enzyme loading and spatial organization. Stability issues are overcome through postimmobilization polymer coating. The general applicability of this heterogeneous biocatalyst is demonstrated by its scale-up in both batch and packed bed reactors, allowing a product yield of >80%. The continuous process is fed with H_2_O_2_ as the oxygen source, reaching a space-time yield (STY) of 0.76 g·L^–1^·h^–1^, maintained for the first 12 h. Finally, this flow system is telescoped with a second plug-flow reactor packed with a different heterogeneous biocatalyst integrating an additional transaminase. As a result, this 6-enzyme 2-reactor system sequentially transforms 1,ω-diols into 1,ω-amino acids while *in situ* recycling NAD^+^, depleting H_2_O_2_, and generating O_2_.

## Abstract 2

Controlling the spatial organization of coimmobilized 5/6 enzymes on porous microbeads allows the intensification of the transformation of 1,ω-diols into 1,ω-hydroxy/amino acids.

## Introduction

Cell-free biocatalysis is gaining momentum in manufacturing value-added chemicals, especially when dealing with multistep reaction cascades.^1−3^ As enzymes often exhibit exquisite selectivity under mild reaction conditions, they are very promising catalysts to be incorporated into synthetic routes for more efficient and sustainable chemical manufacturing.^4^ Multienzyme cascades allow us to carry out multistep chemical reactions (simultaneously or sequentially) in one pot, avoiding isolation and purification during the process.^4^ Furthermore, the control of the activity ratio in enzyme cascades minimizes intermediate inhibitory effects and side product accumulation that may jeopardize the overall throughput of the cascade.^5^ However, the stability of enzyme cascades under industrial operational conditions is normally compromised when free enzymes are involved. To overcome this issue, enzyme immobilization emerges as a great solution to enhance enzyme stability and allows enzyme recycling once the reaction is completed.^6,7^ Moreover, immobilized enzymes present greater versatility to be implemented in different types of reactors. Not surprisingly, enzyme immobilization is the key enabling technology revolutionizing flow biocatalysis.^8−13^

Besides stabilization, enzyme immobilization can also tune the spatial organization of enzymes across the three-dimensional (3D) structure of the immobilization supports. Achieving precise spatial rearrangement of enzymes within solid supports is critical for optimizing the performance of any immobilized multienzyme system. The strategic colocalization of enzymes may maximize the efficient mass transfer of intermediates and cofactors between various active sites. For example, NADH oxidases immobilized on the outer surface of porous supports present higher activity than those immobilized on the inside due to lower mass transport restrictions to molecular oxygen.^14^ Furthermore, another successful example is the coimmobilization of multienzyme systems that demand the recycling of cofactors *in situ* and/or the removal of byproducts.^15^ However, spatial organization and colocalization of several enzymes imply their coimmobilization on the same support, which is not trivial as enzymes with different physicochemical properties may need different immobilization chemistries. In this context, supports activated with different reactive groups, also named heterofunctional supports, have been successfully applied for the coimmobilization of multienzyme systems.^15,16^ The vast majority of heterofunctional supports offer the combination of only two reactive groups: one (i.e., ionic, hydrophobic, metal chelate groups) to drive the enzyme adsorption and the other (i.e., epoxy, aldehyde, glyoxyl, and vinyl groups) to react with the exposed nucleophilic residues on the enzyme surface to form covalent and irreversible bonds.^17−20^ The combination of these two groups allows a two-step enzyme immobilization, in which the enzyme is first absorbed very rapidly (close contact) and then irreversibly attached to the support. As most multienzyme systems are composed of multimeric enzymes, the coimmobilization of enzymes on pre-existing heterofunctional supports is unable to fully stabilize their quaternary structure. To that aim, postimmobilization techniques based on the enzyme polymer coating are a recurrent strategy to avoid subunit lixiviation of the immobilized enzymes during the operation. The gain in stability when an immobilized enzyme is coated with a polymer brush is widely reported in the literature not only for single enzymes^21^ but also for multienzyme systems.^15,16^

**Scheme 1:**
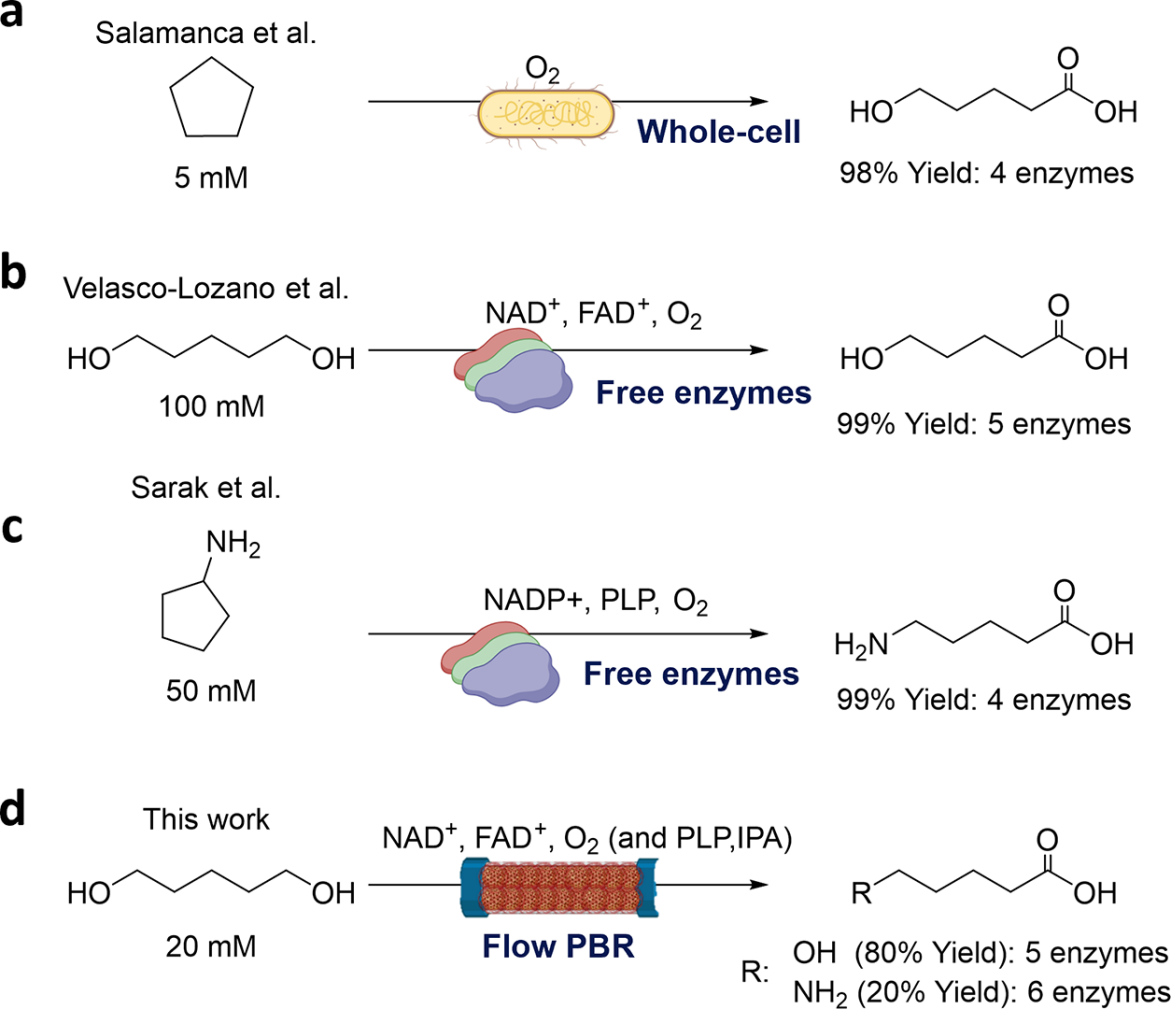
Different Catalytic Approaches for the Synthesis of 5-Hydroxy (5-HP) or 5-Aminopentanoic (5-AP) Acid (a) Using resting cells in batch. (b, c) Using free enzymes in batch. (d) Using immobilized multienzyme systems operated in flow.

The recently developed cell-free biosynthetic cascade that transforms 1,ω-diols into ω-hydroxy acids (ω-HA) is an excellent candidate to benefit from the coimmobilization of the multienzyme system through tuning its intraparticle spatial organization.^22^ In this 5-enzyme cascade, two NAD^+^-dependent dehydrogenases (ADHs) synergistically catalyze the double oxidation of 1,ω-diols to their corresponding lactones that are subsequently hydrolyzed by a lactonase (LAC) to yield the target ω-hydroxy acids. The efficiency of the process relies on the *in situ* recycling of NAD^+^ driven by an NADH oxidase (NOX) that concomitantly produces hydrogen peroxide, a harmful oxidant that is removed by catalase (CAT) to avoid enzyme inactivation. Previous work proposes a similar biosynthetic pathway toward the synthesis of ω-HA from cyclopentane but using resting cells as the enzyme chassis. In the whole-cell biotransformation, the maximum product titer is 5 mM concentration of ω-HA (Scheme 1a).^23^ In contrast, our cell-free system can increase the product titer 20 times with superior green and sustainable metrics (Scheme 1b).^22^ Nevertheless, the incompatibility between the immobilization chemistries needed for each enzyme forced us to heterogenize the system using two different supports where the biosynthetic cascade was physically segregated, with one of the dehydrogenases far away from the NAD^+^ recycling system. This segregation yielded a lower product titer than the system in solution and presented limited reusability as the product yield dramatically decreased after the first operational cycle.^22^ Other cell-free systems have accomplished the transformation of cyclic alkyl amines into ω-amino acids through a sequential process coupling transaminases and oxidoreductases (Scheme 1c).^24^

In this work, we have heterogenized the 5-enzyme system described in Scheme 1b using a trifunctional support (AG-Co^2+^/A/G) developed by our group.^25^ This support has proven successful in individually immobilizing the five enzymes involved in the biocascade, achieving active and stable heterogeneous biocatalysts for most of them. Enzymes can be immobilized through His-tag coordination, ionic adsorption, and covalent bonds, as this support displays cobalt chelates, positively charged secondary amines, and aldehyde (from glutaraldehyde) groups, respectively. Upon kinetic characterization, the coimmobilized multienzyme system was optimized by tuning the intraparticle enzyme spatial distribution, finding the key role of the NOX localization for the overall productivity and stability of the cascade. Finally, the multifunctional heterogeneous biocatalyst was further stabilized by postimmobilization polymeric coating and increasing the NOX/CAT loads. The optimal solid biocatalyst was submitted to a one-pot transformation of 1,5-pentanediol (1,5-PD) as a model diol to yield 5-hydroxypentanoic (5-HP) acid in consecutive batch cycles, demonstrating excellent operational stability and scalability. Finally, we packed this heterogeneous multienzyme system in a plug-flow column to set a packed bed reactor for which we optimized the oxygen source to maximize both the product titer and space-time yield (Scheme 1d). Ultimately this packed bed reactor was telescoped to another one containing an immobilized transaminase for the continous production of 5-aminopentanoic acid (5-AP).

## Results and Discussion

### Optimization of the Spatial Organization in Multifunctional Heterogeneous Biocatalysts

**Figure 1:**
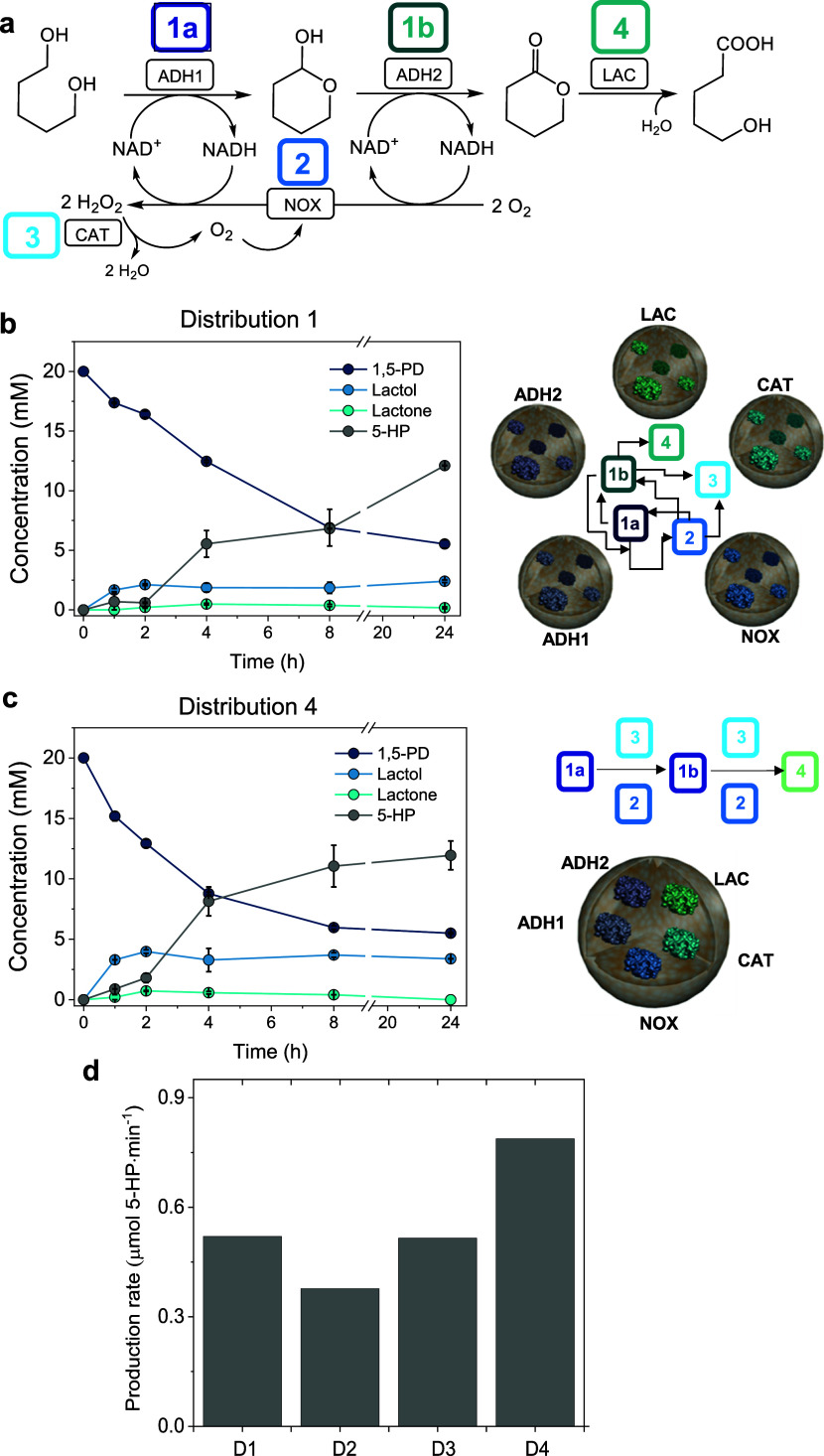
Effect of the spatial organization on the performance of different HBs for the batch biosynthesis of 5-HP. (a) Reaction scheme. Reaction time courses catalyzed using distribution 1 (b), individually immobilized enzymes in different particles, or distribution 4 (c), a coimmobilized multienzyme system on the same particle. In the right panels, the interparticle (b) or the intraparticle (c) diffusion of the intermediates between the different enzymes. Data in (b, c) represent the mean value and standard deviation (error bars) of two independent experiments. (d) Maximum production rate of 5-HP achieved with different multifunctional heterogeneous biocatalysts with different spatial distributions (D1–D4). The maximum production rate was calculated in the first 4 h of the reaction course. In all cases, the reaction mixture consisted of 20 mM 1,5-PD, 1 mM NAD^+^, and 0.15 mM FAD^+^ dissolved in 100 mM sodium phosphate buffer, pH 8. The employed FAD^+^ concentration was based on a previously reported optimization where the NOX enzyme displays the highest specific activity.^28^

The enzyme cascade is composed of two NAD^+^-dependent alcohol dehydrogenases from *Bacillus stearothermophilus* (ADH1)^26^ and the horse liver (ADH2)^27^ to synergistically oxidize 1,5-PD to δ-valerolactone (lactone) via its corresponding lactol intermediate (tetrahydro-2H-pyran-2-ol). As mentioned above, the pool of NAD^+^ is replenished by an oxygen-dependent NADH oxidase from *Thermus thermophilus* HB27 (NOX)^28^ coupled to a catalase from the bovine liver (CAT) that depletes the hydrogen peroxide generated as a byproduct of NOX. Finally, a lactonase from *Sulfolobus islandicus* (LAC)^29^ hydrolyzes δ-valerolactone (tetrahydro-2H-pyran-2-one) to 5-HP (Figure 1a). In previous work, this multienzyme system showed very promising conversion yields by optimizing the enzyme ratio to overcome the bottleneck of the reaction (the oxidation of the lactol into the lactone).^22^ However, we encountered issues when trying to scale up the reaction (from 1.5 to 25 mL) due to enzyme instability. To overcome these issues, we coimmobilized the five free enzymes involved in the cascade on the trifunctional support described above (AG-Co^2+^/A/G) following different spatial configurations (Table 1). While ADH1 and LAC are immobilized through their His-tags reacting with the cobalt chelates, CAT and ADH2 establish electrostatic interactions with the positively charged secondary amines of the carrier, and NOX forms covalent bonds between its surface lysines and the aldehyde groups displayed at the carrier surface. First, we individually immobilized all enzymes on the same support, resulting in five monofunctional heterogeneous biocatalysts (HB1 to HB5). In this configuration, each enzyme is immobilized on a bead different from the others, naming this spatial distribution as D1 (Entry 1, Table 1). Second, ADH1 and ADH2 were immobilized separately on AG-Co^2+^/A/G but coimmobilized with NOX and CAT, resulting in the biocatalysts HB6 and HB7, respectively, and finally mixed with LAC immobilized on AG-Co^2+^/A/G by its own (HB5) to assemble configuration D2 (Entry 2, Table 1). Third, NOX and CAT were coimmobilized with both ADHs (ADH1 and ADH2) on AG-Co^2+^/A/G, yielding the heterogeneous biocatalyst HB8 that was mixed with HB5 containing only LAC to assemble configuration 3, D3 (Entry 3, Table 1). Finally, the five enzymes were sequentially coimmobilized on AG-Co^2+^/A/G to prepare biocatalyst HB9 with configuration 4, D4 (Entry 4, Table 1). All HBs were incubated with 1 M glycine upon 2 h of enzyme immobilization to block the remaining aldehydes, which did not participate in the enzyme attachment.

**Table 1:** Immobilization Parameters of Enzymes Bound to AG-Co^2+^/A/G with Different Spatial Distributions, Enzyme Loads, and Polymer Coatings

entry	distribution	heterogeneous biocatalyst	enzyme	enzyme load (mg·g^–1^)	Ψ^a^ (%)	recovered activity (U·g^–1^)/(%)^b^
1	D1	HB1	ADH2	15^c^	100	0.42 (23)
		HB2	NOX	0.41	81	0.67 (5)
		HB3	CAT	0.010^c^	40	107 (25)
		HB4	ADH1	5	100	1.52 (11)
		HB5	LAC	1.49	98	0.35 (21)
2	D2	HB6	ADH2	7.4^c^	99	0.5 (26)
			NOX	0.21	33	0.21 (15)
			CAT	0.004	56	109.7 (26)
		HB7	ADH1	2.45	98	1.19 (28)
			NOX	0.12	94	0.67 (5)
			CAT	0.004	65	127.3 (30)
		HB5	LAC	1.49	98	0.35 (21)
3	D3	HB8	ADH2	4.20^c^	99	na (na)
			NOX	0.13	57	0.40 (20)
			CAT	0.004	38	237 (61)
			ADH1	1.26	100	0.66 (23)
		HB5	LAC	1.49	98	0.35 (21)
4	D4	HB9	ADH2	3.0^c^	100	na (na)
			NOX	0.12	65	0.53 (30)
			CAT	0.0023	19	343 (36)
			ADH1	1.0	100	0.9 (50)
			LAC	0.30	85	0.33 (46)
5	dD5	HB10	ADH2	3.0^c^	100	na (na)
			NOX	0.14	79	0.50 (26)
			CAT	0.12^c^	99	104 (8)
			ADH1	0.99	99	1.08 (50)
			LAC	0.32	100	0.18 (23)
6	dD5	HB11	ADH2	3.0^c^	100	na (na)
			NOX	0.15	82	0.51 (26)
			CAT	0.12^c^	99	120 (9)
			ADH1	1.0	100	1.04 (47)
			LAC	0.32	100	0.18 (23)
7	dD5	HB12	ADH2	3.0^c^	100	na (na)
			NOX	0.66	74	1.25 (17)
			CAT	0.87^c^	73	770 (35)
			ADH1	1.0	100	1.07 (49)
			LAC	0.32	100	0.20 (25)
8	dD5	HB13	ADH2	3.0^c^	100	na (na)
			NOX	0.72	80	1.7 (20)
			CAT	1.14	95	116 (5)
			ADH1	1.0	100	1.5 (53)
			LAC	0.32	100	0.26 (32)

a Immobilization yield, Ψ = (immobilized activity/offered activity) × 100.

b (%) Recovered activity of the immobilized enzyme is defined as the coefficient between the specific activity of the immobilized enzymes and the specific activity of the soluble ones.

c Total protein content in a semipurified enzyme extract.

d D5 is the distribution where the five enzymes are coimmobilized and colocalized at the outer region of the same bead. All data herein presented correspond to the mean value of three independent enzyme activity and protein concentration assays. In all cases, the standard deviation was never higher than 5% of the mean value.

These four spatial distributions imply that intermediates must follow different interparticle diffusion pathways toward the final product. Accordingly, in D1, all intermediates must travel from one particle to the other to be processed by their corresponding enzyme (Figure 1b). In contrast, as D2 segregates each oxidation step but confines the NAD^+^ recycling and H_2_O_2_ removal, the only intermediates forced to travel between particles are lactol and lactone (Figure S1a). In the case of D3, only lactone must diffuse between different particles to be hydrolyzed by LAC (Figure S1b). Finally, as D4 confines the five enzymes inside the same particle, interparticle transport of intermediates is not needed to complete the cascade target product (Figure 1c). Expectedly, the immobilization parameters for each enzyme varied depending on whether the enzymes were individually immobilized or coimmobilized together (Table 1). This phenomenon was already reported for ADH1, whose recovered activity when immobilized alone is different from the activity recovered when coimmobilized with other enzymes.^30^

Once the nine different HBs (HB1–9) were prepared, they were mixed to assemble the multienzyme systems with the corresponding spatial distribution (D1–D4) in the reaction, keeping a protein mass ratio of 1:3:0.18:0.012:0.32 for ADH1:ADH2:NOX:CAT:LAC. We selected this protein mass ratio because it resulted in the highest yield of ω-HA when the cascade was catalyzed by the same free enzymes.^22^ Monitoring the reaction courses, we observed that D1 and D4 converted 75% of 1,5-PD (16.3 mM), yielding up to 60% of 5-HP (11.8 mM) in 24 h (Figure 1b,c), whereas D2 and D3 only reached a 32% (6.4 mM) and 35% (7.2 mM) 5-HP yield, respectively, after the same time (Figure S1a,b). Remarkably, Figure 1d shows that all enzymes coimmobilized on the same particle (Entry 4, Table 1) transform 1,5-PD into 5-HP 1.6 times faster than all enzymes physically segregated into different particles (Entry 1, Table 1). Since the oxidation of the lactol intermediate is the rate-limiting step in this cascade due to the high apparent *K*_M_ of ADH2 toward it,^31^ its greater accumulation using the HB9 with the D4 configuration may speed up the lactone production, thus contributing to improving the overall throughput of the cascade when using this spatial configuration. As the D1 and D4 configurations present the most promising results, in terms of product yield and productivity, we discarded the D2 and D3 configurations for further studies.

### Operational Stability of the Heterogeneous Multifunctional Biocatalyst with Different Spatial Configurations

**Figure 2:**
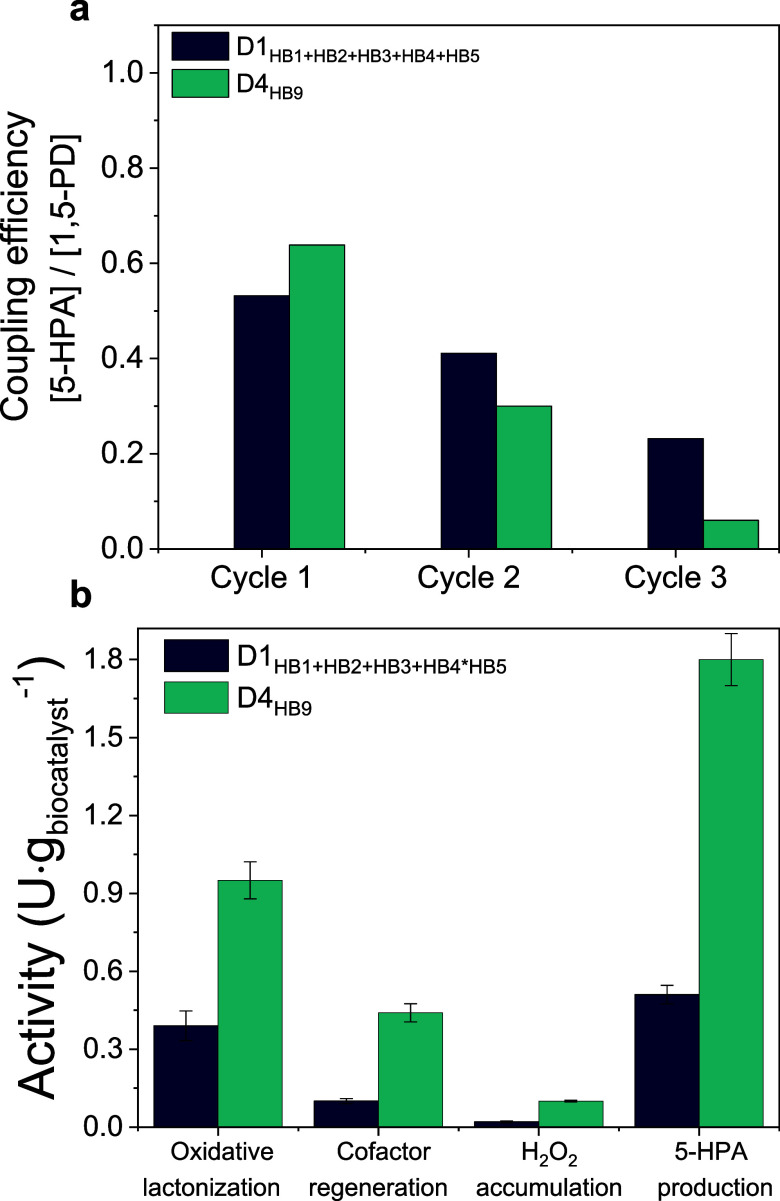
Effect of the enzyme system distribution on the performance of HBs for batch 5-HP biosynthesis. (a) Coupling efficiency after consecutive batch cycles performed with HBs with distributions D1 and D4. (b) Individual activities of different reaction steps determined spectrophotometrically for HBs with distributions D1 and D4 (see the Supporting Information for more details). Data in panel (b) correspond to the mean value and standard deviation (error bars) of triplicate activity assays.

Due to the promising performance of HBs under D1 and D4 spatial configurations, we tested their operational stability in consecutive batch reaction cycles by assessing the cascade coupling (Figure 2). This latter parameter is defined as the mol of 5-HP produced per mol of 1,5-PD consumed, where the ideal system would reach a coupling efficiency value of 1, indicating a perfect cascade orchestration, where the substrate (1,5-PD) and intermediates (lactol and lactone) are quantitatively converted into the final target product (5-HP). Although HB9 with configuration D4 (Entry 4, Table 1) outperforms HB1–5 in configuration D1 (Entry 1, Table 1) for the first cycle, the cascade coupling efficiency was higher with D1 than with D4 in the second and third consecutive cycles, pointing out that the five enzymes coimmobilized together are less stable than those separately immobilized on the same support (Figure 2a). To understand the lower operational stability of the coimmobilized system (D4), we investigated the catalytic efficiency of each cascade step using a set of spectrophotometric assays that allowed us to determine the activity of the diol oxidation (Figure S2a), the NADH oxidation (Figure S2b), the hydrogen peroxide accumulation (Figure S3), and the ω-hydroxy acid production (Figure S4). Figure 2b shows that HB9 in configuration D4 is 3 and 4 times faster for the diol oxidation and NADH recycling, respectively, than configuration D1 using HB1–5. These results match the reaction time courses (Figure 1), supporting the higher overall throughput of this cascade when it is catalyzed by the five enzymes coimmobilized on the same porous particle of AG-Co^2+^/A/G. However, the D4 spatial configuration accumulates H_2_O_2_ 5 times faster than configuration D1, suggesting that CAT in the coimmobilized system cannot match the activity of NOX. As H_2_O_2_ is a liaison for enzymes, its accumulation in the reaction catalyzed by HB9 in configuration D4 explains the system inactivation during the process. Despite the unsatisfactory operational stability, the high efficiency of the coimmobilized biocatalysts encouraged us to enhance its operational stability by optimizing its capacity to remove the H_2_O_2_ formed *in situ*, without limiting the NADH recycling.

### Optimization of the Intraparticle Spatial Distribution and Loading of the 5-Enzyme Coimmobilized System to Maximize Its Performance

**Figure 3:**
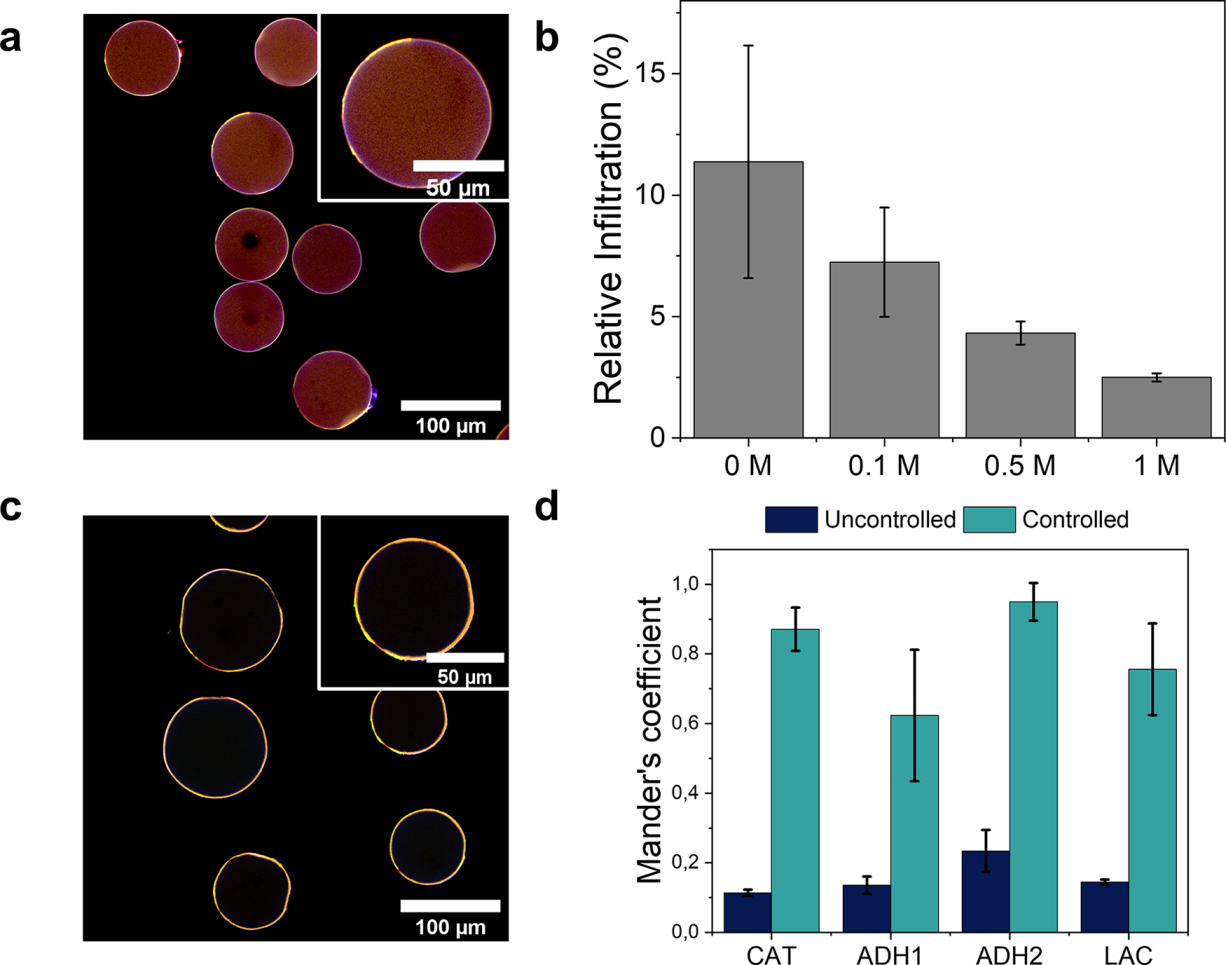
Intraparticle spatial organization of the enzymes. (a) Merged CLSM images of a 5-enzyme system coimmobilized in the absence of NaCl (HB9). (b) Relative infiltration of NOX through the radius of AG-Co^2+^/A/G beads when immobilized at different NaCl concentrations (HB10). (c) Merged CLSM images of a 5-enzyme system coimmobilized in the presence of 1 M NaCl (HB10). (d) Mander’s coefficient of rhodamine-labeled NOX overlapping each other labeled enzyme. Uncontrolled (without salt). Controlled (with 1M NaCl). This coefficient denotes the fraction of NOX that colocalizes with each of the other enzymes. In (b, d), data represent the mean value and standard deviation (error bars) of at least 10 microbeads. For CLSM, enzymes were labeled with different fluorophores as follows: ADH1 (RhB, λ_ex_: 561 nm, orange color), ADH2 (Atto 488, λ_ex_: 488 nm, green color), LAC or CAT (Atto 390, λ_ex_: 405 nm, blue color), and NOX (A647, λ_ex_: 633 nm, red color).

To investigate why hydrogen peroxide is accumulated when the cascade is catalyzed by HB9 in configuration D4, we studied the intraparticle spatial distribution of a coimmobilized enzyme system by confocal laser scanning microscopy (CLSM) using enzymes labeled with compatible fluorophores for colocalization studies. Figures 3a and S5 show that four of the coimmobilized enzymes are located at the outer surface of the particle, whereas NOX is located at the inner regions of the beads. This spatial distribution agrees with the spatial distribution found for the five enzymes individually immobilized on this support (Figure S6), as previously reported.^25^ The intraparticle segregation of NOX and CAT may explain their impaired activities. Thus, hydrogen peroxide can accumulate during biotransformation as it is produced at large distances (inner regions of the particle) from where it can be removed (outer regions of the particle), damaging the coimmobilized enzymes.

To improve the hydrogen peroxide removal, we optimized the spatial distribution of NOX by tuning its immobilization kinetics, colocalizing all five enzymes at the outer surface of the beads. As previously demonstrated, high enzyme immobilization rates yield immobilized enzymes located at the outer parts of microbeads,^32^ while slowly immobilized enzymes are uniformly distributed across the beads. The immobilization rate can be easily controlled by either adding immobilization competitors or modifying the immobilization buffer and/or conditions. To favor a more rapid NOX immobilization rate and enable its localization at the outer surface of the beads, we performed its immobilization on AG-Co^2+^/A/G in the presence of a gradient of NaCl concentration (0, 0.1, 0.5, and 1 M).

1 M NaCl was needed to locate NOX at the outer surface of the support, colonizing the outer 2% radius of the beads (2.5 μm on average) (Figures 3b and S7). Figures 3c and S8 show the CLSM images that demonstrate the colocalization of the five enzymes at the outer region of the same bead. When 1 M NaCl is added to the immobilization buffer, Mander′s coefficients of NOX regarding the other enzymes (Figure 3d, Table S1) determined from the CLSM images confirm that NOX colocalizes with the rest of the enzymes to a higher extent than when NaCl is not added. As the support is positively charged, it can repel NOX and slow its immobilization. Hence, we hypothesize that the chlorides will act as counterions to the positive amine groups of AG-Co^2+^/A/G, minimizing the repulsion and consequently immobilizing NOX faster on the outermost surface. The outer localization of NOX brings it closer to CAT, enhancing their cooperative action but also increasing the NAD^+^ recycling efficiency as the oxygen transport from the bulk to NOX is facilitated. The HB bearing the five enzymes colocalized at the outer surface of the beads will be now referred to as HB10 with distribution 5 (D5) (Entry 5, Table 1). Furthermore, we corroborated that this new spatial location of NOX negligibly affects the immobilization pattern of the other enzyme members of the cascade (Figure S8).

**Figure 4:**
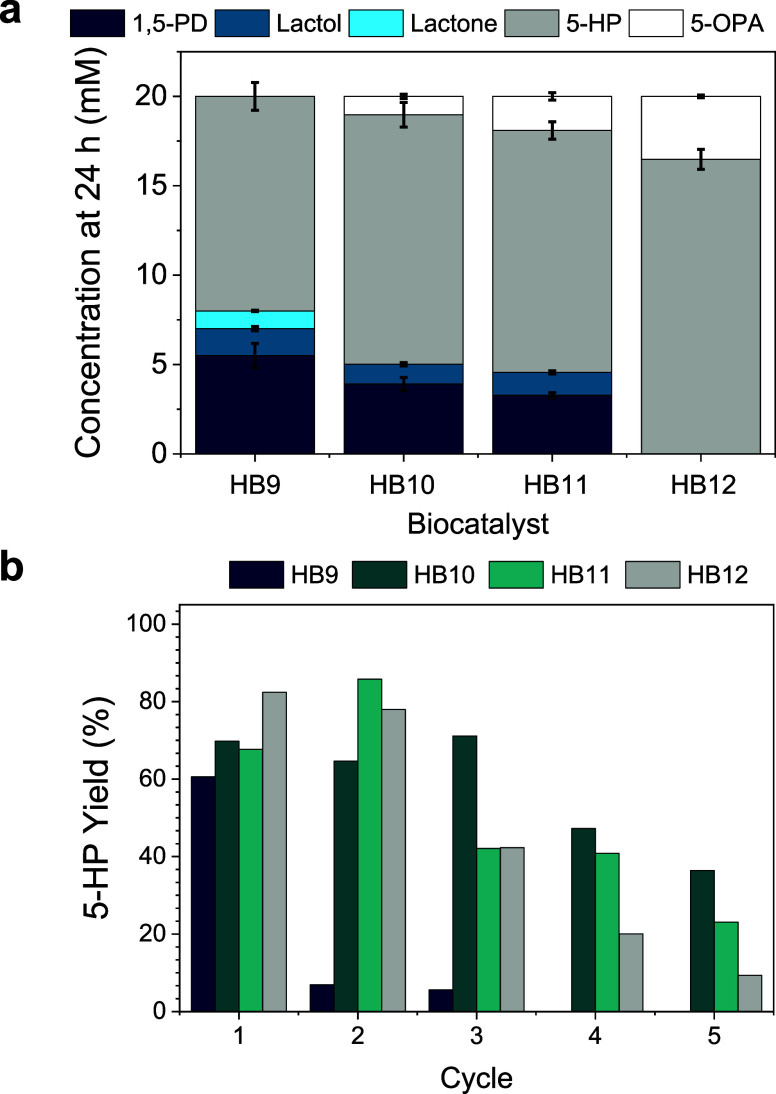
Effect of the NOX spatial distribution, immobilization time, and enzyme loading on the performance of different HBs for 5-HP biosynthesis in batch. (a) 5-HP titer after 24 h of reaction using HB9 (NOX in), HB10 (NOX out), HB11 (NOX out, 16 h of immobilization), and HB12 (higher loadings of NOX(out)/CAT). (b) 5-HP yield after consecutive 24 h batch cycles using HB9-HB12. Reaction mixture: 20 mM 1,5-PD, 1 mM NAD^+^, 0.15 mM FAD^+^, and 200 mM sodium phosphate buffer pH 8 at 30 °C. Data in (a) correspond to the mean value and standard deviation (error bars) of two independent experiments.

Next, we evaluated the effect of the intraparticle NOX spatial distribution on biocatalyst productivity (Figure 4a) and operational stability (Figure 4b). First, we observe that the localization of NOX at the outer surface of the beads increases the 5-HP titer upon 24 h of reaction and maintains the chromatographic product yield (CY ≈ 70%) constant for three consecutive batch cycles unlike HB9 (Entry 4, Table 1) where NOX is localized in the deeper surface of the porous support (Figure 4b). Unfortunately, HB10 suffered operational inactivation in the fourth operational cycle, observing a CY decay of 20%. To further increase the operational stability of HB10, we incubated the immobilized enzymes for longer times (16 h at 4 °C) before the blocking step to fabricate HB11 (Entry 6, Table 1). Longer immobilization times pursue promoting the formation of more attachments between the residues at the enzyme surface and the aldehydes of AG-Co^2+^/A/G, to ultimately improve the enzyme stability as reported elsewhere.^20^ Nevertheless, the increase in the immobilization time enhances neither the efficiency nor the operational stability of HB11. Finally, to further optimize the performance of the HB10 biocatalyst, we increased the load of NOX and CAT by 4.7 and 7.25 times, respectively, resulting in a heterogeneous biocatalyst named HB12 with distribution D5 (Entry 7, Table 1). The specific activity of the immobilized NOX in HB12 decreased 1.8 times due to the higher protein density within the porous beads. Previous studies support the fact that NOX is less catalytically efficient at high protein loads, suggesting that protein crowding negatively affects the performance of this enzyme.^14^ Despite this activity reduction, HB12 converted 100% 1,5-PD to yield 80% 5-HP (16 mM) after 24 h. Surprisingly, we observed 20% production (4 mM) of the 5-oxopentanoic acid (5-OPA), indicating the overoxidation of the target 5-HP. This product overoxidation hints at a very efficient NAD^+^-recycling system that boosts the oxidative activity of the two coimmobilized dehydrogenases (ADH1 and ADH2). This overoxidation is mainly attributed to HLADH, which can catalyze the intermediate oxidation of 6-hydroxycaproic acid into 6-oxohexanoic acid during the synthesis of 6-aminocaproic acid (6ACA) from caprolactone.^45^ Regarding the operational stability, the excess of immobilized CAT drove to a less operationally stable biocatalyst as the product yield dramatically decayed to 10% upon reusing this biocatalyst in five consecutive batch cycles. In summary, the overall efficiency and operational stability of the 5-enzyme heterogeneous biocatalyst are optimized by localizing NOX at the outer surface of the beads and increasing the NOX and CAT loading in the biocatalyst, yet longer immobilization times negligibly improve the biocatalyst performance. To note, higher enzyme loads resulted in an HB being less operationally stable. To understand whether the operational inactivation of HB12 relies on enzyme lixiviation due to an excessive load, we performed an sodium dodecyl-sulfate polyacrylamide gel electrophoresis (SDS-PAGE) analysis of HB10–12. This electrophoretic analysis reveals that enzyme lixiviation similarly occurred in all of them (Figure S9); thus, operational inactivation may be triggered by the enzyme subunit leaching (quaternary structure disassembly) under reaction conditions, among other causes.

### Improvement of the Multifunctional Heterogeneous Biocatalysts by Cationic Polymer Coating

As most immobilized enzymes on HB12 are lixiviated during their operational use, we decided to stabilize their quaternary structure by polymer coating using polyallylamine (PAH). The enzyme coating with this cationic polymer enhances the performance of dehydrogenases and oxidases as previously reported by our group.^33^ To that aim, after sequentially coimmobilizing the five enzymes with the optimal spatial distribution and enzyme loading, we coated them with PAH, fabricating a new version of HB12 named HB13 (Entry 8, Table 1). The primary amines of PAH react with the remaining aldehyde groups of the support not involved in enzyme attachment, acting as an ionic macromolecular cross-linker of enzyme subunits and as a blocking agent for those remained aldehydes. This polymer coating increased the recovered activity of ADH1, ADH2, and NOX, suggesting that the aminated polymer has a stabilizing effect on the quaternary structure of the immobilized enzymes. SDS-PAGE analysis (Figure S9, lanes 9 and 10) confirms the stabilizing effect since enzyme subunits coated with PAH are lixiviated to a lower extent after consecutive batch cycles.

**Figure 5:**
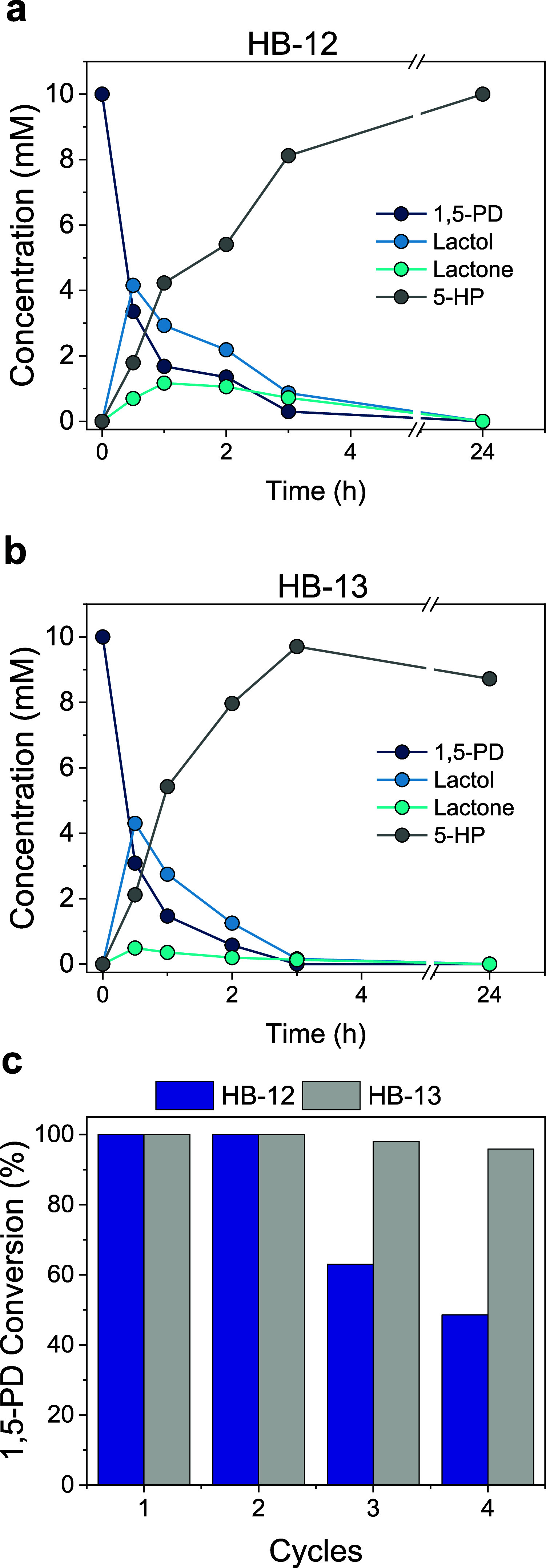
Effect of the PAH coating on the performance of HBs for batch 5-HP biosynthesis. (a) Reaction time courses using HB12 (without the PAH coating) and (b) HB13 (with the PAH coating). (c) Batch operational stability of HB12 and HB13 in consecutive cycles of 24 h. All reaction mixes contained 10 mM 1,5-PD, 1 mM NAD^+^, 0.15 mM FAD^+^, and 200 mM sodium phosphate buffer pH 8 at 30 °C. Data in all panels correspond to the mean value and standard deviation (error bars) of two independent experiments.

Next, we tested HB13 for the stepwise oxidation/hydrolysis of 1,5-PD into 5-HP in one pot. As a result, HB13 achieves a CY of 100% using 10 mM substrate in only 3 h in comparison with the 80% conversion achieved with the same biocatalyst but blocked with glycine (HB12) (Figure 5a,b). When the substrate load was scaled up to 20 mM, HB13 reached 90% CY in 6 h (Figure S10a). Then, we studied the operational stability of HB13 by submitting it to consecutive recycling in 24 h batch cycles. Figure 5c shows how the PAH-coated heterogeneous biocatalyst was operationally stable for four consecutive cycles, while the substrate conversion decayed below 50% when using the noncoated HB12. This tendency is also reflected in the 5-HP yield along the cycles (Figure S10b). Interestingly, we observed that HB13 maintains the 5-OPA yield after four consecutive cycles, whereas HB12 was unable to produce such an overoxidized product in any analyzed cycle, as expected from its lower oxidation capacity (Figure S10c).

After successfully assembling a productive and stable multifunctional heterogeneous biocatalyst (HB13), we scaled the batch reaction volume up to 30 mL with 3.3% (w:v) biocatalyst load and monitored the product titer, the oxygen concentration, and the pH along the reaction course (Figure S11). As pH decay occurs concomitantly with 5-HP production, we manually kept the pH constant to a value of 8 by NaOH titration during the operation. The performance of HB13 in this scaled cascade is notorious as we achieved 80% 5-HP yield after 96 h of operation, which means a product titer of 16.4 mM (ca. 0.5 mmol), a maximum volumetric productivity of 0.053 g·L^–1^·h^–1^, and a specific mass productivity (MP) of 1.1 mg·g_HB13_^–1^·h^–1^. Furthermore, we observed a 6% reduction in the oxygen saturation in the reaction bulk during the first hour of the reaction. This oxygen depletion is related to the high rate of the first oxidative step (1,5-PD to lactol) associated with a very efficient NAD^+^ regeneration system that concomitantly consumes molecular oxygen by the action of NOX. Then, the oxygen level increases until it reaches its steady saturation concentration (22%). This experimental evidence supports a very efficient coupling between ADH1 and its cofactor regeneration system (NOX/CAT).

### Implementation of the Optimal Multifunctional Heterogeneous Biocatalyst in Packed Bed Reactors for Continuous Synthesis of 5-HP

In our efforts to intensify the process, we integrated HB13 into a packed bed reactor (PBR). This PBR packed with 1 g of HB13 was first flushed with 10 mM 1,5-PD at 0.02 mL·min^–1^, showing no product formation. The UV–vis spectra of samples collected from the PBR outlet demonstrated that the pool of the redox cofactor was NADH (Figure S12), indicating the premature cascade halt due to inefficient NAD^+^ recycling. Interestingly, the outlet samples were colorless, indicating that FAD^+^ was either absorbed to the surface of HB13 as reported for other heterogeneous biocatalysts coated with cationic polymers or reduced to FADH_2_ but not reoxidized due to the absence of oxygen.^34^ This latter hypothesis is supported by the poor solubility of oxygen in aqueous medium (0.25 mM) and the lack of aeration within the PBR, explaining why the PBR fails to transform 1,5-PD into 5-HP due to the inefficient FAD^+^ and NAD^+^*in situ* regeneration. To overcome such a limitation, we flushed the PBR with an air-saturated solution, but unfortunately, we did not detect the product.

**Figure 6:**
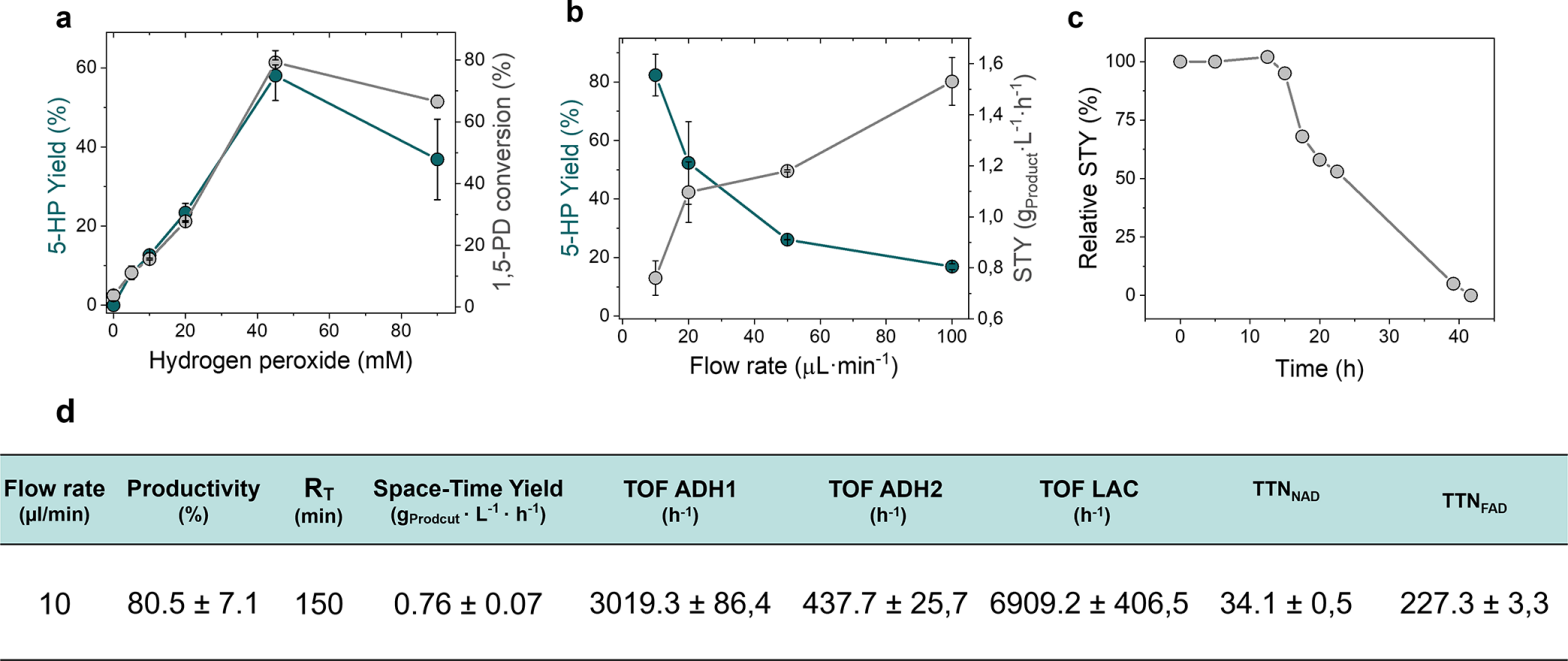
Synthesis of 5-HP in flow catalyzed by the PBR packed with HB13. (a) Scheme of the packed bed reactor. (b) Product yield and substrate conversion at supplying a range of H_2_O_2_ concentrations. (c) 5-HP yield and STY at different flow rates. (d) Evolution of STY along the operational time. Relative STY is defined as the percentage of STY regarding the initial STY at different times on the stream. (e) Table of operational parameters under the optimal conditions. Turnover frequency (TOF) is defined as the moles of substrate converted per moles of enzyme per hour, with 1,5-PD being the substrate for ADH1, lactol being the substrate for ADH2, and lactone being the substrate for LAC. Total turnover numbers (TTNs) of cofactors (NAD^+^ and FAD^+^) are defined as the molar concentration of 5-HP x 2 divided by the molar concentration of each corresponding cofactor at the maximum STY. Data in panels (b, c, e) represent the mean value and the standard deviation (error bars) of two independent experiments. Data in (c) correspond to one operation run.

Inspired by previous work from Nidetzky’s^35^ and Turner’s^36^ groups who managed to release soluble oxygen in a flow reactor flushing hydrogen peroxide in the presence of catalase, we decided to follow a similar approach to enhance NAD^+^ recycling driven by NOX (Figure 6a). As HB13 integrates CAT, we run the PBR with this multifunctional heterogeneous biocatalyst using 20 mM 1,5-PD and varying H_2_O_2_ concentration at 0.01 mL·min^–1^ (Figure 6b). At 45 mM H_2_O_2_, we achieved a maximum substrate conversion and product yield (CY) of 80 and 60%, respectively, determined by gas chromatography (GC). However, at 90 mM H_2_O_2_, we observed a dramatic decay in the CY likely due to the harmful effect of hydrogen peroxide on enzyme stability.^37,38^ As expected, we also observed a linear correlation between the pH drop and the product titer at the reactor outlet due to the accumulation of higher concentrations of the target ω-hydroxy acid (Figure S13). To yield 20 mM product, the cascade demands 40 mM O_2_ within the PBR. To reach this oxygen concentration, the flow reactor must be pressurized up to 30 bar according to the Henry law.^39,40^ By feeding 45 mM H_2_O_2_ and 20 mM 1,5-PD, we theoretically supply the PBR with 42.5 mM oxygen (22.5 mM directly from the fed H_2_O_2_ and 20 mM produced during the NAD^+^ recycling) through the catalase-driven disproportionation of H_2_O_2_. In contrast, the same PBR fed with O_2_ dissolved in reaction (aqueous) medium under ambient pressure and 25 °C is approximately 0.25 mM. Thus, the use of H_2_O_2_ as an oxygen supplier improves the safety of the process.

Once the optimal H_2_O_2_ concentration was found, we next challenged the PBR to a flow rate ramp to determine its productivity limits. Figure 6c shows that the maximum product CY is achieved at the lowest flow rate, giving as a result the lowest STY. In contrast, the highest STY productivity occurred at a flow rate of 0.1 mL·min^–1^ at the expense of product titer with a CY as low as 18%. Thus, we selected 0.01 mL·min^–1^ and 45 mM H_2_O_2_ as the optimal conditions to operate the PBR. Under these conditions, we achieved 80% 5-HP in 150 min (residence time) with STY = 0.76 ± 0.07 g·L^–1^·h^–1^ and a specific mass productivity of 0.76 mg·g_HB13_^–1^·h^–1^ (Figure 6c). This latter parameter is slightly lower than that achieved with the 30 mL batch reactor under the same reaction conditions, suggesting that the oxygen supply is still more efficient in a stirred tank than in an H_2_O_2_-fed PBR. Moreover, the ^1^H NMR of the sample collected directly from the outlet of the PBR shows a purer product than the sample separated from the batch process catalyzed by the free-enzyme system (Figure S14). Under the optimal reaction conditions described above, the PBR was continuously operated for 44 h (Figure 6d). The STY was maintained during the first 12 h of operation and, afterward, steadily decreased to 0 after 44 h of operation, detecting no product in the reactor outlet. *Postused* and *ex situ* activity assays revealed that the ADH activity of 44 h-operated HB13 was 2 times lower than that of its fresh counterpart. In contrast, NOX retained 90% of its initial activity in the exhausted biocatalysts. Therefore, the STY decay during the continuous operation is linked to the inactivation of the coimmobilized ADH1 and ADH2. This inactivation may be explained by an inefficient disproportionation of H_2_O_2_ due to the exhaustion of the catalase, accumulating H_2_O_2_ in the PBR. This accumulation can drive enzyme inactivation^37,38^ and carrier modification (i.e., cobalt chelates^41^ or amine oxidation^42^) that ultimately limit the long-term operational stability of the multifunctional heterogeneous biocatalyst. This inactivation effect has been also observed in batch reactors using coimmobilized oxidases and catalases after several controlled additions of exogenous H_2_O_2_.^35^

Additionally, we calculated the turnover frequency (TOF) of each reaction step by analyzing the profiles of products and intermediates at the PBR outlet. Figure 6e shows that the rate-limiting step is the intermediate oxidation of lactol to lactone, as the TOF of ADH2 is 7- and 16-fold lower than that of ADH1 and LAC, respectively. Finally, the total turnover numbers (TTNs) of the cofactors were also determined, demonstrating that each molecule of NAD^+^ and FAD^+^ can be utilized up to 34 and 237 times, respectively, by ADHs and NOX.

**Figure 7:**
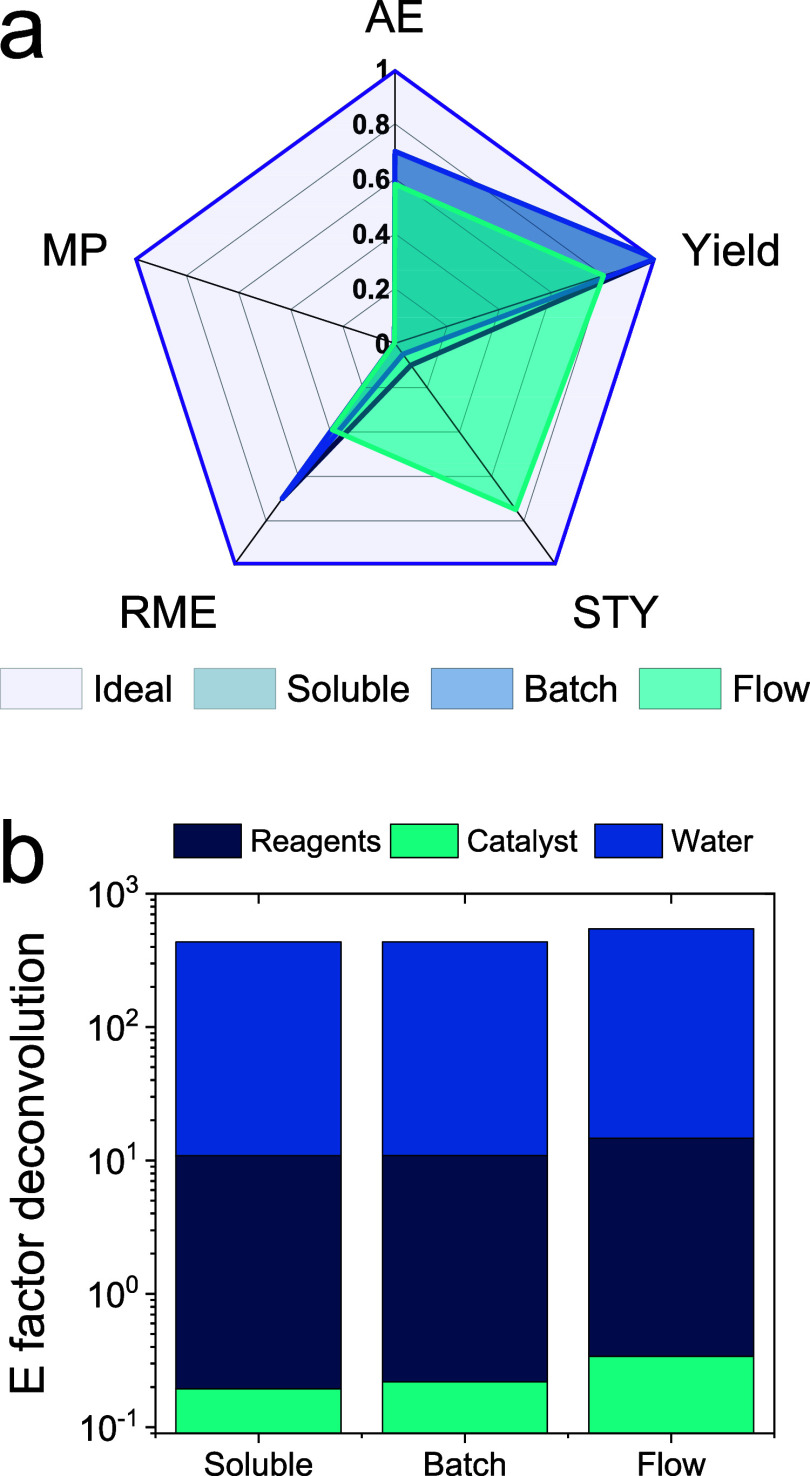
Green metric parameters of soluble and heterogeneous biocatalysts in the biosynthesis of 5-HP from 1,5-PD. (a) AE = atom economy; MP = mass productivity; RME = reaction mass efficiency; STY = space-time yield (ideal STY corresponds to 1 g·L^–1^·h^–1^, which is the minimum STY for high-priced products). (b) E factor deconvolution. Detailed reactor operation details are given in Table S2.

Finally, we assessed the green and sustainability metrics of our heterogeneous biocatalysts (working in batch and flow conditions) to compare them with their soluble counterparts (Figure 7). To that aim, we calculated the reaction mass efficiency (RME) and mass productivity (MP) defined as the mass of the product divided by either the mass of reactants or the total mass including catalysts and solvents, respectively. Second, we calculated the atom economy (AE) and the spatial time yield (STY) for the three compared systems. To this regard, we considered an ideal STY equal to 1 g·L^–1^·h^–1^, which is the minimum STY for high-priced products (Figure 7a).^43^ The flow reactor improves the STY up to 7-fold compared to the batch systems but presents lower RME and AE due to the addition of H_2_O_2_. In all three systems, MP is the weakest parameter due to the large excess water required to achieve this biotransformation. Finally, we assessed the total E factor for the three reactor configurations (Figure 7b). All systems attained very similar E factors, indicating similar sustainability. It is worth mentioning that in the three cases, 97% of the E factor corresponds to water. Particularly, the contribution of the biocatalyst is 150% higher in the flow reactor than in batch configurations using either free or immobilized enzymes.

### Implementation of Two Telescoped PBRs for the Biotransformation of 1,5-PD into 5-Aminopentanoic (5-AP) Acid

**Figure 8:**
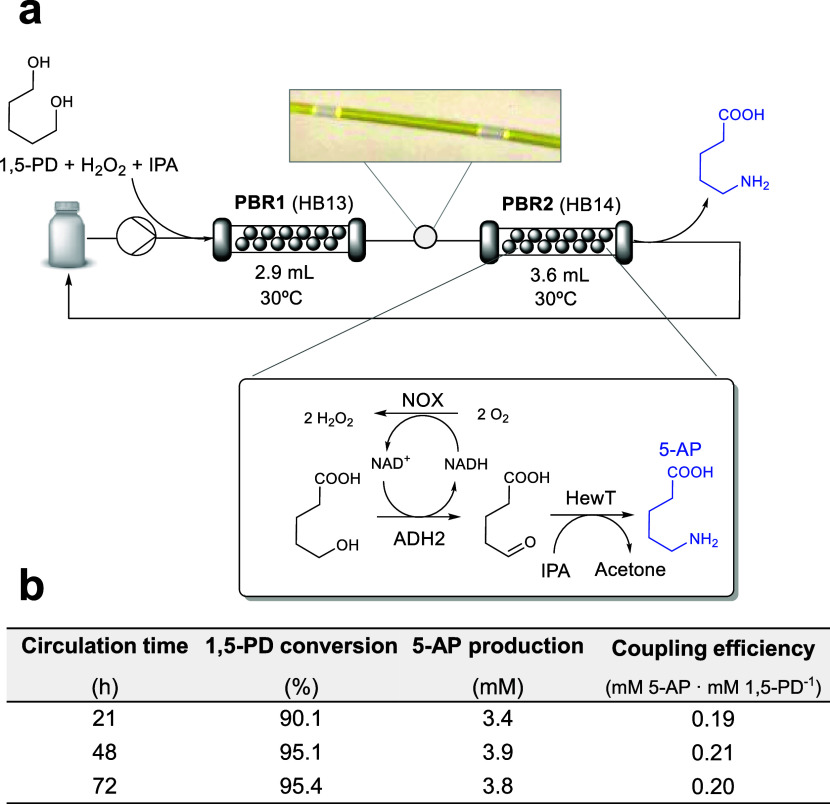
Continuous-flow synthesis of 5-AP by telescoped packed bed reactors. (a) Scheme of the telescoped flow reaction setup. The enzymatic cascade carried out in PBR1 is depicted in Figure 1a. PBR1 containing HB13 (1.7 g) was connected to PBR2, which was packed with HB14 (2.7 g). The flow biotransformation was performed in circulation mode (flow rate: 0.02 mL·min^–1^) for 72 h. A segmented air–liquid flow occurred naturally between PBR1 and PBR2 due to oxygen produced by PBR1. (b) Time course of the telescoped reaction in flow. The reaction mix contained 20 mM 1,5-PD, 2 mM NAD^+^, 1 mM FAD^+^, 45 mM H_2_O_2_, 10 mM isopropyl amine (IPA), and 0.1 mM PLP in 100 mM sodium phosphate buffer pH 8. Data in (b) correspond to one operation run.

To further exploit the potential of this multifunctional heterogeneous biocatalyst, we studied its application in the biosynthesis of 5-aminopentanoic (5-AP) acid. This ω-amino acid has gained attention for its potential use in nylon synthesis.^44,45^ To achieve this, we combined HB13 with a previously reported multifunctional heterogeneous biocatalyst (HB14)^45^ for the conversion of 5-hydroxypentanoic (5-HP) acid into 5-AP (Figure 8a). We assembled HB14 by coimmobilizing ADH2, NOX, and a transaminase from *Halomonas elongata* (HewT) on methacrylate beads. This support was functionalized with epoxy and aldehyde groups for the irreversible immobilization of ADH2, NOX, and HewT and further coated with poly(ethylenimine) (PEI) to improve the biocatalyst stabilization (Figure S15a). In contrast to our previous work,^45^ we selected here an H_2_O_2_-forming NADH oxidase (NOX) encouraged by its excellent behavior as part of HB13 in flow reactors fed with H_2_O_2_. Immobilization yields were higher than 75% for all coimmobilized enzymes, whereas the recovered activities ranged from 3.5 to 65% depending on the enzyme, with NOX being the one that recovered the lowest activity (Figure S15b).

Once HB14 was fabricated, we packed HB13 and HB14 in two different PBRs and telescoped them for the continuous biotransformation of 1,5-PD into 5-AP (Figure 8a). Then, the continuous-flow reaction was carried out in circulation mode at 0.02 mL·min^–1^, allowing the unreacted substrates to contact both PBRs for longer times. Remarkably, no additional air supply was needed for PBR2 as the segmented air–liquid flow was naturally generated from PBR1 by the action of CAT (Figure 8a, inlet). Isopropyl amine (IPA) was added to the reaction mixture as an amine donor for the last transamination reaction. Under these conditions, 95% of 1,5-PD was consumed and 3.5 mM 5-AP was produced in 21 h (Figure 8b), which mean three reactor cycles for PBR1 and PBR2, with a total residence time of 7.25 and 9 h, respectively. Longer reaction times (up to 72 h) failed to increase the 5-AP titer, mainly due to the inactivation of the immobilized ADHs, whose activity decayed by more than 70% upon their continuous operation (Table S3). To enhance the efficiency of this 6-enzyme 2-reactor system for the complete conversion of 1,5-PD into 5-AP in flow, we anticipate ongoing efforts. These include replacing ADHs with more robust ones, refining the immobilization strategy, and increasing the excess of amine donors.

## Conclusions

In this work, we have immobilized a 5-enzyme cascade into porous supports as an artificial chassis to transform 1,ω-diols into either ω-hydroxy acids in one pot through a concurrent manner. Through a holistic approach and a deep characterization of the fabricated heterogeneous biocatalysts, we achieve a multifunctional heterogeneous biocatalyst with excellent reusability and capacity to be integrated into flow reactors. To that aim, we first tuned the spatial organization of the five enzymes involved in the cascade. Our findings indicate that the optimal spatial configuration is having the five enzymes coimmobilized on the same support bead and colocalized at its outermost region. The resulting heterogeneous biocatalyst underwent further optimization to enhance the efficiency of the NAD^+^-recycling system with H_2_O_2_ removal *in situ*. Additionally, a cationic polymer coating was applied to the coimmobilized enzyme system, stabilizing their protein quaternary structures. This optimization journey resulted in a multifunctional heterogeneous biocatalyst denoted as HB13, which demonstrates excellent performance and operational stability. Specifically, HB13 was productive in the biosynthesis of 5-hydroxypentanoic (5-HP) acid from 1,5-pentanediol (1,5-PD) under both discontinuous and continuous operations in batch and flow reactors, respectively.

In the transition of HB13 to packed bed reactors (PBRs) for continuous-flow processing, we encountered a challenge of inefficient reactor aeration under atmospheric pressure. To overcome this limitation, we introduced H_2_O_2_ into the reactor to generate *in situ* the oxygen required by the NAD^+^-recycling system driven by the NADH oxidase–catalase pair. The optimal spatial configuration and robustness of these immobilized enzymes enable the utilization of up to 45 mM H_2_O_2_ within the HB13 porous particles. The flow transformation of diols into 5-HP reaches similar yields as those reached with whole-cell and cell-free systems transforming cyclic alkanes^23^ and amines^24^ into 5-HP. Volumetric productivities were not compared because the studies were performed at different substrate concentrations. Nonetheless, we made the effort to determine the green metrics of the optimized heterogeneous biocatalyst operated in batch and flow reactors. The continuous process results in higher productivity but slightly lower values of atom economy and mass productivity due to the exogenous supply of H_2_O_2_. Finally, HB13 packed into a PBR was telescoped with another PBR containing three coimmobilized enzymes, leading to the successful conversion of 1,5-PD into 5-aminopentanoic acid (5-AP). This achievement, involving a total of six different enzymes spatially organized in two PBRs, sets a record for the number of enzymes (6) and reaction steps (7) assembled in a cell-free biosynthetic cascade operating in flow. Despite the involvement of oxygen-dependent enzymes, our approach demonstrated reasonable productivity.

Our work highlights the potential of strategically arranging immobilized enzymes to create more productive and complex heterogeneous biocatalytic systems, paving the way for the continuous biosynthesis of industrially relevant products. While the current system, with seven reactions catalyzed by six enzymes in two telescoped packed bed reactors, may not be considered a complete cell-free metabolic pathway, we anticipate that further advancements in rational enzyme coimmobilization will contribute to expanding the capabilities of continuous chemical biomanufacturing, enriching the biomanufacturing portfolio.

## Methods

### Materials

The enzymes, alcohol dehydrogenase from *B. stearothermophilus* (ADH1), reduced nicotinamide adenine dinucleotide (NADH) oxidase from *T. thermophilus* HB27 (NOX), the lactonase from *S. islandicus* (LAC), and the ω-transaminase from *H. elongata* (HewT) were produced as previously reported.^25,46^ 6% Cross-linked agarose (AG) beads (particle size: 50–150 μm; pore diameter: 300 nm) were purchased from Agarose Bead Technologies (Madrid, Spain). ReliSorb EP400/SS was acquired from Resindion S.R.L. (Binasco, Italy). Compounds such as ethylenediamine (EDA), imidazole, iminodiacetic acid, cobalt chloride, sodium periodate, sodium hydroxide, Rhodamine B isothiocyanate, Atto 390 NHS ester, Atto 488 NHS ester, sodium acetate, sodium chloride, sodium phosphate, sodium bicarbonate, glutaraldehyde (GA), poly(allylamine) (*M*_w_: 65,000 *g* mol^–1^) (PAH), poly(ethylenimine) (*M*_w_: 60,000 g mol^–1^) (PEI), Ampliflu Red, protein gel stain, 1,5-pentanediol (1,5-PD), tetrahydro-2H-pyran-2-ol (lactol), δ-valerolactone (lactone), 5-hydroxypentanoic (5-HP) acid, alcohol dehydrogenase from the horse liver (ADH2), pyridoxal-5′-phosphate (PLP), and *S*-methylbenzyl amine (S-MBA) were acquired from Sigma-Aldrich Chemical Co. (St. Louis, IL). Alexa Fluor 647 NHS ester was purchased from Fisher Scientific. All other reagents were analytical grade.

### Enzyme Activity Assays

Enzyme activities were spectrophotometrically measured in transparent 96-well microplates with a flat bottom (Nunc), employing a Microplate Reader Epoch2 (BioTek Instruments) provided with software Gen5.

#### ADH1 and ADH2 Activity

200 μL of a reaction mixture containing 10 mM 1,5-PD and 1 mM NAD^+^ in sodium phosphate buffer at pH 8 was incubated with 5 μL of enzymatic solution or 10 μL of immobilized enzyme suspension (properly diluted) at 30 °C. The increase in the absorbance at 340 nm due to the reduction of NAD^+^ was recorded. One unit of activity was defined as the amount of enzyme that was required to reduce 1 μmol NAD^+^ to NADH per minute under the assayed conditions.

#### NOX Activity

200 μL of a reaction mixture containing 0.2 mM NADH and 150 μM flavin adenine dinucleotide (FAD^+^) in 50 mM sodium phosphate buffer pH 8 at 30 °C was incubated with 5 μL of enzymatic solution or 10 μL of immobilized enzyme suspension (properly diluted) at 30 °C. The oxidation of NADH was monitored as a decrease in the absorbance at 340 nm. One unit of activity was defined as the amount of enzyme that was required to oxidize 1 μmol NADH to NAD^+^ per minute under the assayed conditions.

#### CAT Activity

200 μL of a reaction mixture containing 35 mM hydrogen peroxide in 100 mM sodium phosphate pH 8 at 30 °C was incubated with 5 μL of the enzymatic solution or 10 μL of immobilized enzyme suspension (adequately diluted). The catalase activity was measured by recording the decrease in absorbance at 240 nm. One unit of CAT activity was defined as the amount of enzyme required for the disproportionation of 1 μmol hydrogen peroxide per minute under the assessed conditions.

#### LAC Activity

Lactonase activity was indirectly monitored by a decrease in pH triggered by the formation of 5-HP from its corresponding lactone hydrolysis. Briefly, 200 μL of a reaction mixture containing 1 mM δ-valerolactone, 0.1% acetonitrile (ACN), and 0.25 mM p-nitrophenol in 2.5 mM sodium phosphate buffer at pH 7.0 was incubated with 5 μL of enzymatic solution or 10 μL of an immobilized enzyme suspension (properly diluted) at 30 °C. The decrease in the absorbance of p-nitrophenol (pH indicator) at 410 nm was recorded. One unit of activity was defined as the amount of enzyme that was required to produce 1 μmol 5-HP (titrated by pH change) per minute under the assayed conditions.

#### HewT Activity

5 μL of enzyme solution (0.5 mg·mL^–1^) or 10 μL of immobilized enzyme suspension (1:20 w/v) was incubated with a 200 μL mixture containing 2.5 mM *S*-MBA, 2.5 mM pyruvate, and 0.1 mM PLP in 50 mM potassium phosphate buffer pH 8.0 at 30 °C. Transaminase activity was monitored by recording the increase in absorbance at 245 nm for 5 min.

### Synthesis of the Triheterofunctional Support (AG-Co^2+^/A/G)

We prepared AG functionalized with GA, EDA, and IDA/cobalt groups (AG-Co^2+^/A/G) as described elsewhere.^25^ Briefly, we prepared epoxy-activated agarose (AG-E), and then, we activated it with iminodiacetic acid (AG-E/IDA) by preparing a suspension of 10 g of AG-E in 100 mL of 0.5 mM iminodiacetic acid at pH 11 under gentle agitation at 200 rpm for 1 h at room temperature (RT). After filtering and rinsing with 10 volumes of water, AG-E/IDA was incubated with 10 volumes of 1 M ethylenediamine at pH 11 under gentle agitation at 200 rpm and room temperature overnight (AG-E/IDA/A). Afterward, the support was filtered and washed with 10 volumes of water and then incubated overnight with a 15% glutaraldehyde solution in 200 mM sodium phosphate buffer pH 7 (AG-IDA/A/G) under gentle agitation at 200 rpm at room temperature. Subsequently, after filtering and washing, the support was incubated with 10 volumes of 30 mg·mL^–1^ CoCl_2_ for 2 h at room temperature (AG-Co^2+^/A/G). Finally, the support was filtered and washed with abundant water and stored at 4 °C protected from light.

### Optimization of the Spatial Organization in a Heterogeneous 5-Enzyme System

#### HB1–HB9

The assembly of heterogeneous biocatalysts was conducted by mixing 1 g of AG-Co^2+/^A/G with 10 mL of each enzyme solution in 100 mM sodium phosphate buffer at pH 7, achieving different enzyme loads as shown inTable 1. Depending on the spatial distribution, the immobilization sequence differed (*vide infra*). In all cases, the enzyme–support suspension was maintained under gentle agitation at 25 rpm and 4 °C for 2 h. Subsequently, a blocking step was done by adding 10 mL of 1M glycine at pH 8 to 1 g of the immobilized biocatalysts by adding 1. The suspension was incubated for 16 h at 25 rpm and 4 °C in a rotatory shaker. Once the immobilization was blocked, it was washed five times with five volumes of 25 mM sodium phosphate buffer, pH 8, filtered, and stored at 4 °C. Specifically, for biocatalysts HB1 to HB5, each enzyme was individually immobilized. For coimmobilized systems, the immobilization process was conducted stepwise in the following order: HB6: 1° NOX/CAT and 2° ADH1; HB7: 1° NOX/CAT and 2° ADH2; HB8: 1° NOX/CAT, 2° ADH1, and 3° ADH2; and HB9: 1° NOX/CAT, 2° ADH1, 3° ADH2, and 4° LAC. Between enzyme immobilization steps, immobilized samples were washed three times with five volumes of 25 mM sodium phosphate buffer pH 8 and filtered.

After the immobilization process, we calculated the immobilization yield (Ψ) corresponding to the amount of the immobilized enzyme(s) on the solid support as described in eq 1
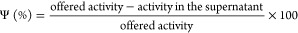
1where offered activity is the initial activity of the soluble enzyme which was incubated with the support and activity in the supernatant is the enzyme activity found in the filtrate of the enzyme suspension upon immobilization.

Likewise, we calculated the total and relative recovered activity of the immobilized enzyme(s) by calculating the total recovered activity per gram of solid support and the percentage of specific recovered activity (rRA (%)) resulting after the immobilization process according to eq 2.
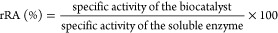
2

#### HB10

The assembly of a heterogeneous biocatalyst was conducted under the same conditions described above but in the case of NOX. After immobilizing all enzymes following the order 1° NOX in the presence of NaCl (0–1 M), 2 °CAT/ADH2, 3° ADH1, and 4° LAC, finally, a blocking step was done by the addition of glycine (1 M, pH 8) followed by soft agitation overnight at 25 rpm and 4 °C.

#### HB11–HB13

The assembly of HB11, HB12, and HB13 was conducted as for HB10 with a slight variation in the protocol. In the case of HB11, after the immobilization of the five enzymes, the immobilizate was incubated at 4 °C overnight in sodium phosphate buffer pH 8; then, it was blocked with 1 M glycine at 4 °C for 3 h. In the case of HB12, we followed the same protocol as HB10 but with loading 4 and 7 times more NOX and CAT, respectively (see entry 7, Table 1). In HB13, after the 5-enzyme immobilization, the immobilizate was incubated at 4 °C overnight with 10 mg·mL^–1^ polyallylamine (PAH) in 25 mM HEPES buffer at pH 8.

### Preparation of HB14

3 g of EP400/SS microbeads were incubated with 30 mL of 0.1 M H_2_SO_4_ for 30 min. After filtration and washing with H_2_O, 30 mL of 10 mM NaIO_4_ were added and the suspension was incubated for 2 h. After filtration and washing with H_2_O, a 30 mL mixture containing 12 mg of ADH2 (protein concentration determined by Bradford assay) and 15 mg of NOX in 0.1 M sodium bicarbonate buffer at pH 10.0 was added. The suspension was incubated for 1 h on ice. Then, 30 mg of NaBH_4_ were added and the suspension was incubated for 30 min on ice. After filtration and washing with H_2_O, 30 mL of 0.3 M EDA in 0.1 M sodium bicarbonate buffer at pH 8.5 was added, and the suspension was incubated for 2 h. After filtration and washing with H_2_O, 30 mL of a solution containing 15 mg of HewT in 50 mM potassium phosphate buffer at pH 8 was added. The suspension was incubated for 5 h on ice. Finally, 30 mL of a solution of 10 mg·mL^–1^ PEI (*M*_w_ 60,000 mol g^–1^) was added to block the remaining epoxy groups, and the suspension was incubated for 16 h on ice.

### Colorimetric Assays to Independently Monitor Intermediate Reaction Steps in the Cascade

#### Oxidative Lactonization

Oxidative lactonization was monitored, as shown in Figure S2a. Briefly, 200 μL of a reaction mixture containing 10 mM 1,5-PD and 1 mM NAD^+^ in 100 mM sodium phosphate buffer at pH 8 was incubated with 10 μL of a suspension of HBs (properly diluted) at 30 °C. The increase in the absorbance at 340 nm due to the reduction of NAD^+^ was recorded. One unit of activity is defined as the reduction of 1 μmol NAD^+^ to NADH per minute under the assayed conditions.

#### Cofactor Regeneration

Cofactor regeneration was monitored, as shown in Figure S2b. Briefly, 200 μL of a reaction mixture containing 0.2 mM NADH and 0.15 mM FAD^+^ in 100 mM sodium phosphate buffer at pH 8 was incubated with 10 μL of a suspension of HBs (properly diluted) at 30 °C. The increase in the absorbance at 340 nm due to the reduction of NAD^+^ was recorded. One unit of activity is defined as the oxidation of 1 μmol NADH to NAD^+^ per minute under the assayed conditions.

#### Hydrogen Peroxide Accumulation

Hydrogen peroxide accumulation was monitored as shown in Figure S3.^47^ Briefly, 200 μL of a reaction mixture containing 0.5 μg·mL^–1^ HRP, 20 mM 1,5-PD, 1 mM NAD^+^, 0.15 mM FAD^+^, and 50 μM Ampliflu Red in 100 mM sodium phosphate buffer at pH 8 was incubated with 10 μL of a suspension of HBs (properly diluted) at 30 °C. The increase in the absorbance at 560 nm due to the formation of resorufin was recorded. One unit of activity is defined as the oxidation of 1 μmol Ampliflu Red per minute at the assayed conditions.

#### ω-Hydroxy Acid Production

ω-Hydroxy acid production was monitored as shown in Figure S4.^48^ Briefly, 200 μL of a reaction mixture containing 20 mM 1,5-PD, 1 mM NAD^+^, 0.15 mM FAD^+^, and 0.1 mM Cresol Red in 2.5 mM sodium phosphate buffer at pH 8 was incubated with 10 μL of a suspension of HBs (properly diluted) at 30 °C. The decrease in the absorbance at 580 nm due to the decrease in the pH was recorded. Also, the absorbance at 340 nm was recorded at the same time to guarantee that there is no NADH accumulation since it decreases the pH. One unit of activity is defined as 1 μmol of H^+^ (carboxylic group) produced per minute at the assayed conditions.

### Enzyme Labeling with Fluorescent Probes

Fluorescent labeling of ADH1, ADH2, LAC, and CAT was done using a methodology reported elsewhere.^49^ Each enzyme solution in 100 mM sodium bicarbonate buffer at pH 8.5 (ADH1: 0.2 mg·mL^–1^, ADH2: 1 mg·mL^–1^, LAC: 1 mg·mL^–1^, CAT: 3.9 mg·mL^–1^, and NOX: 1.2 mg·mL^–1^) was mixed with the respective fluorophore: Rhodamine B (ADH1), Atto 488 (ADH2), Atto 390 (LAC and CAT), and A647 (NOX) at 1:1 molar ratio (stocks of each fluorophore were prepared in DMSO). The labeling reaction was then incubated for 2 h under gentle shaking at 25 °C. Later, buffer exchange and removal of unreacted fluorophores were done by filtering the enzyme solution through a tangential ultrafiltration unit (10 kDa) equilibrated in 25 mM sodium phosphate-buffered solution at pH 7.

### Confocal Laser Scanning Microscopy (CLSM) Imaging

The localization and distribution of fluorophore-labeled immobilized enzymes for the different distributions were recorded with a Spectral ZEISS LSM 880 confocal microscope. Imaging was performed using 20× (0.8 NA) and 40× (immersion: water, 1.2 NA) objectives and different excitation lasers, λ_ex_: 405 nm for Atto 390, λ_ex_: 488 nm for Atto 488, λ_ex_: 561 nm for Rhodamine B, and λ_ex_: 633 nm for A647. All samples of each biocatalyst with the fluorescently labeled immobilized enzyme were suspended in an 8-well chamber slide (Ibidi) in a 1:200 (w:v) buffered suspension in 25 mM phosphate buffer at pH 7. The resulting micrographs were analyzed with FIJI software^50^ to determine the relative infiltration radius and the colocalization parameters.^51^

### Batch Reactions to Transform 1,5-PD into 5-HP Catalyzed by Different HBs

100 mg of HB19 or a mix of HB1–8 was placed inside a capped plastic tube (5 mL) containing 300 μL of a reaction mixture consisting of 20 mM 1,5-PD, 1 mM NAD^+^, and 0.15 mM FAD^+^ in 100 mM sodium phosphate buffer pH 8, allowing atmospheric oxygen supplementation by punching the cap with an open needle. Reaction mixtures were incubated at 30 °C and 250 rpm inside an orbital incubator. The reaction course was monitored by withdrawing samples at periodic intervals, which were analyzed by chromatographic methods. In some experiments, this reaction was scaled up. To do so, 1 g of HB13 was placed inside a glass vessel (100 mL) containing 30 mL of a reaction mixture consisting of 20 mM 1,5-PD, 1 mM NAD^+^, and 0.15 mM FAD^+^ in 100 mM sodium phosphate buffer pH 8, allowing atmospheric oxygen supplementation by agitation with impeller blades. The reaction mixture was incubated at 30 °C at 600 rpm. Oxygen and pH were monitored continuously using an oximeter OXROB10 (Pyroscience, Aachen DE) and a pH meter (Mettler Toledo, Columbus), respectively. The reaction progress was monitored by withdrawing samples at periodic intervals and analyzing them with chromatographic methods.

### Operational Stability of HBs

Briefly, 100 mg of HBs was placed inside a capped plastic tube (5 mL) containing 300 μL of a reaction mixture consisting of 10–20 mM 1,5-PD, 1 mM NAD^+^, and 0.15 mM FAD^+^ in 100 mM sodium phosphate buffer pH 8, allowing atmospheric oxygen supplementation by punching the cap with an open needle. Reaction mixtures were incubated at 30 °C at 250 rpm inside an orbital incubator, and samples were collected at 24 h. After each cycle, the HBs were washed once with 10 volumes of 100 mM sodium phosphate buffer at pH 8 and then mixed again with fresh reaction medium to start the following cycle.

### Flow Reactions to Transform 1,5-PD into 5-HP Catalyzed by HB13

The continuous-flow biotransformations were conducted by packing 1 g of HB13 in a plastic plug-flow column and pumping through it a reaction mixture containing 20 mM 1,5-PD, 1 mM NAD^+^, and 0.15 mM FAD^+^ (and additionally 0–90 mM hydrogen peroxide) in 100 mM sodium phosphate buffer pH 8 with a syringe pump at 10–100 μL·min^–1^. Temperature was maintained at 30 °C with a heated bath. The operational performance was monitored by withdrawing samples at periodic intervals and analyzing them by chromatographic and spectroscopic methods (*vide infra*).

### Flow Reactions to Transform 1,5-PD into 5-AP Catalyzed by HB13 and HB14

Flow biotransformations were performed using an R2S/R4 Vapourtec reactor equipped with two PBRs that were composed of an Omnifit glass column (6.6 mm i.d. × 100 mm length) packed with the immobilized enzymes. PBR1 contained 1.7 g of HB13, and PBR2 contained 2.7 g of HB14. First, the system was equilibrated by pumping 100 mM sodium phosphate buffer (pH, 8.0) for 30 min. Second, a reaction mixture containing 20 mM 1,5-PD, 2 mM NAD^+^, 1 mM FAD^+^, 45 mM hydrogen peroxide, 10 mM IPA, and 0.1 mM PLP in 100 mM sodium phosphate buffer pH 8 was passed through the PBRs using an R2S pump. The reaction mixture was pumped at a flow rate of 0.02 mL·min^–1^ at 30 °C until it reached the outlet of the PBR2. At this point, the output was fed back into the recipient containing the reaction mixture, and the reaction was run in circulation mode at 30 °C for 72 h (corresponding to 10.6 passes). Samples were collected after 21, 48, and 72 h and analyzed by GC and High-performance liquid chromatography (HPLC) (*vide infra*).

### High-Performance Liquid Chromatography (HPLC) Analysis

The detection of 5-amino pentanoic acid (5-AP) was performed by FMOC derivatization as previously described.^45^ 50 μL of the biotransformation was added to 100 μL of 100 mM borate buffer at pH 9.0 and 200 μL of 15 mM FMOC in acetonitrile (ACN). After 5 min, 50 μL of the FMOC-derivatized sample was mixed with 200 μL of 0.2% HCl in H_2_O and 200 μL of ACN and filtered with a 0.45 μm poly(tetrafluoroethylene) (PTFE) filter. The samples were analyzed by an HPLC Dionex UltiMate 3000 (Thermo Fisher, Loughborough, U.K.), equipped with a C18 column (3.5 μm, 2.1 × 100 mm) (Waters, Elstree, U.K.). 2 μL of the sample was injected and analyzed using a gradient method, 5:95 to 95:5 (H_2_O/ACN containing 0.1% trifluoracetic acid) over 4 min with a flow rate of 0.8 mL·min^–1^ at 45 °C. The FMOC-derivatized 5-AP (6.37 min) was detected using UV detectors at 265 nm. Molar conversions were calculated with a calibration curve of 5-AP.

### Gas Chromatography (GC) Analysis

(Extraction) Before GC analysis, 50 μL of the reaction sample was mixed with 200 μL of ethyl acetate to perform a liquid–liquid extraction of the compounds of interest containing 2 mM eicosane as an external standard. After the extraction, 30–50 mg of anhydrous MgSO_4_ was added to dry samples before GC analysis.^52^ (Derivatization) All reaction samples were derivatized as described elsewhere.^22^ Samples were derivatized by placing 30 μL of the aqueous reaction in a 1.5 mL Eppendorf tube, followed by the addition of 30 μL of N-methylimidazole and 225 μL of acetic anhydride and incubated by 10 min at room temperature. Afterwards, 300 μL of distilled water were added to the reaction mix and allowed to cool down. Later, liquid-liquid extraction of acetylated compounds was done by the addition of 300 μL of dichloromethane containing 2 mM eicosane as internal standard discarding the aqueous phase. 30–50 mg of anhydrous MgSO_4_ were added to dry samples before GC analysis. Gas chromatography analyses were carried out in an Agilent 8890GC system chromatograph using a J&W HP-5 GC column (30 m × 0.32 mm × 0.25 μm), helium as the support gas, and equipped with a flame ionization detector (FID), with the injector asset at 280 °C and the FID at 300 °C and the injection volume of 1 μL with a split ratio of 10. Separation of extracted compounds in ethyl acetate was done by the following temperature program: the initial temperature at 60 °C, maintained for 2 min, ramp to 160 °C at a rate of 10 °C·min^–1^, ramp 2–240 °C at a rate of 20 °C·min^–1^, and finally maintained for 4 min.

### Nuclear Magnetic Resonance (NMR) Analysis

When specified, reaction samples were analyzed by ^1^H NMR spectra acquired on a Bruker 500 MHz Ultra Shield spectrometer, operating at 500 MHz for ^1^H NMR spectroscopy. Chemical shifts (δ) were reported in parts per million (ppm) and referenced using the residual solvent peak (deuterium oxide; δ = 4.79 ppm). Coupling constants (*J*) were reported in hertz (Hz). The multiplicity of the signals were reported as a singlet (s), doublet (d), doublet of doublets of doublets of doublets (dddd), doublet of quartet (dq), doublet of triplet (dt), triplet (t), and multiplet (m).

## Supporting Information Available

Schemes of colorimetric assays and spatial distribution, supporting CLSM images, SDS-PAGE, 30 mL-scale batch reaction, ^1^H NMRs of products, UV–vis spectra, and raw data of colocalization studies and green metric analysis (PDF)
