# Supplementary material for: Optimized Spatial Configuration of Heterogeneous Biocatalysts Maximizes Cell-Free Biosynthesis of ω-Hydroxy and ω-Amino Acids
Source: ACS Sustain Chem Eng. 2024 Jun 10;12(25):9474–89. doi: 10.1021/acssuschemeng.4c02396 (PMC11394354; doi:10.1021/acssuschemeng.4c02396)
Supplement: Supplementary file 1 — sc4c02396_si_001.pdf [file sc4c02396_si_001.pdf]

Supplementary Information

Optimized Spatial Configuration of Heterogeneous Biocatalysts Maximizes Cell-Free Biosynthesis of ω-Hydroxy and ω-Amino Acids

Javier Santiago-Arcos[a], Susana Velasco-Lozano[a,b,e], Eleftheria Diamanti[a], Ana I. Benítez-Mateos[c,d], Daniel Grajales-Hernández [a], Francesca Paradisi[c], and Fernando López-Gallego[a,f]**

Pages S1-S12

Content

Supplementary Figures .... S 2

Figure S1. Different spatially organized multi-enzyme systems .... S 2

Figure S2. Colorimetric assays for biocatalysts kinetic characterization. .... S 2

Figure S3. Colorimetric assay for measuring the hydrogen peroxide accumulation. .... S 3

Figure S4. Colorimetric assay for measuring the 5-HP production. .... S 3

Figure S5. Uncontrolled spatial distribution of co-immobilized multi-enzyme system in HB9((ADH1-RhB), HB2 (ADH2-ATTO488), HB3 (NOX-A647), HB4 (LAC-Cy3) and HB5 (CAT-Cy3)). .... S 4

Figure S6. Spatial distribution of single immobilized enzymes .... S 4

Figure S7. Control of the spatial distribution of NOX by ionic strength. .... S 5

Figure S8. Controlled spatial distribution of co-immobilized multi-enzyme system .... S 5

Figure S9. SDS-PAGE gels of HBs after five batch cycles. .... S 6

Figure S10. a Reaction-time course of HB13 .... S 6

Figure S11. 30 mL scale batch reaction. .... S 7

Figure S12. UV-Vis Spectra of the collected volume .... S 7

Figure S13. pH values of collected samples after PBR flow reaction .... S 8

Figure S14. ^{1}H NMR of the reaction mixture .... S 8

Figure S15. a Scheme of the immobilization protocol for the preparation of HB14. .... S 9

Supplementary Tables .... S 10

Table S1. JaCoP and Manders coefficients .... S 10

Table S2. Reaction operation conditions are considered for green metric parameters. .... S 10

Table S3. Operational stability of ADH2, NOX, and HewT after the telescoped flow reaction for the synthesis .... S 11

Supplementary References .... S 11

Supplementary Figures

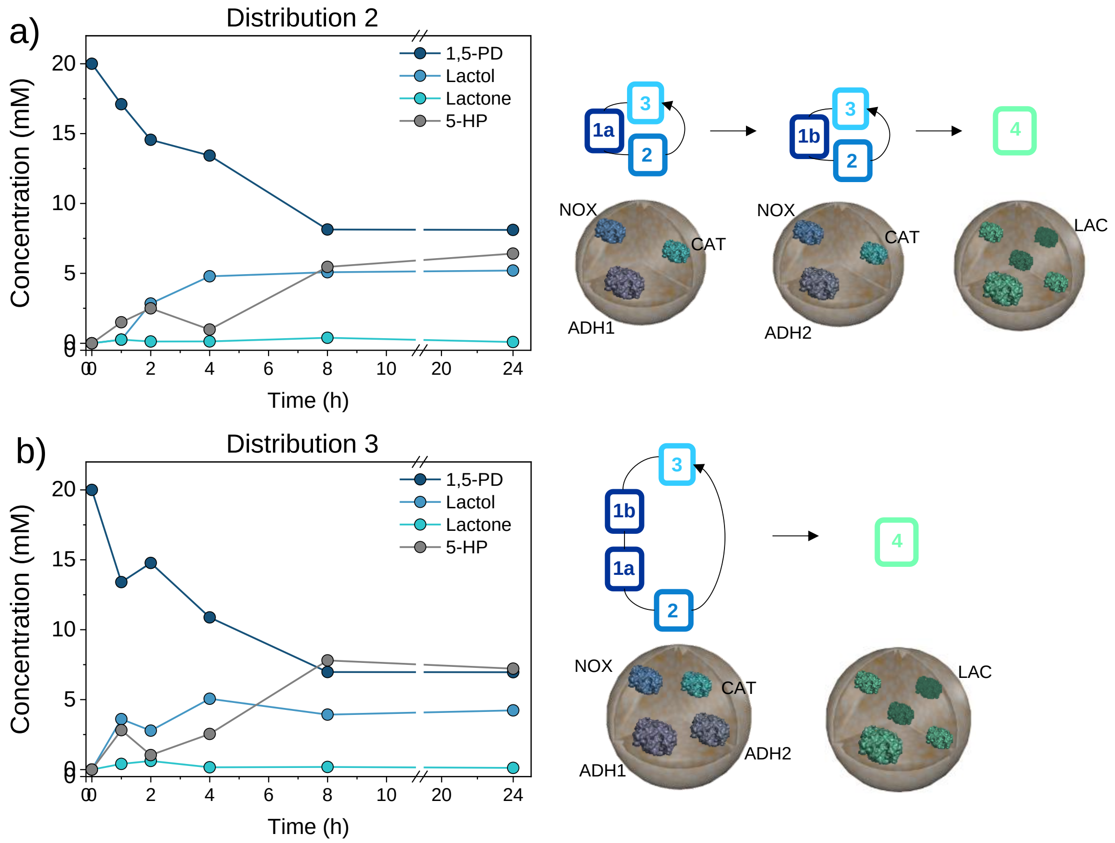

Figure S1. Different spatially organized multi-enzyme systems (distribution 2; D2 and distribution 3; D3) and their catalytic kinetic performance in the synthesis of 5-HP. In all cases, reaction mixtures consisted of 20 mM 1,5-PD, 1 mM NAD^+, and 0.15 mM FAD^+ in 100 mM sodium phosphate buffer pH 8. Distribution 2 is the mixture of HB6, HB7 and HB5. Distribution 3 is the mixture of HB8 and HB5. Data in panels **a** and **b** represent the mean value and standard deviation (error bars) of two independent experiments.

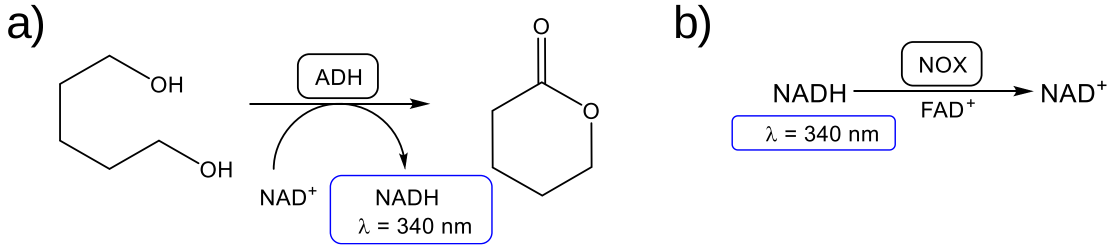

Figure S2. Colorimetric assays for biocatalysts kinetic characterization. a) Oxidative lactonization, recording increment in the absorbance at 340 nm. Reaction conditions: 10

mM 1,5-PD and 1 mM of NAD^+ in 100 mM sodium phosphate buffer pH 8 at 30 ºC. b) Cofactor regeneration, recording increment in the absorbance at 340 nm. Reaction conditions: 0.2 mM NADH, 0.15 mM FAD^+ in 100 mM sodium phosphate buffer pH 8 at 30 ºC.

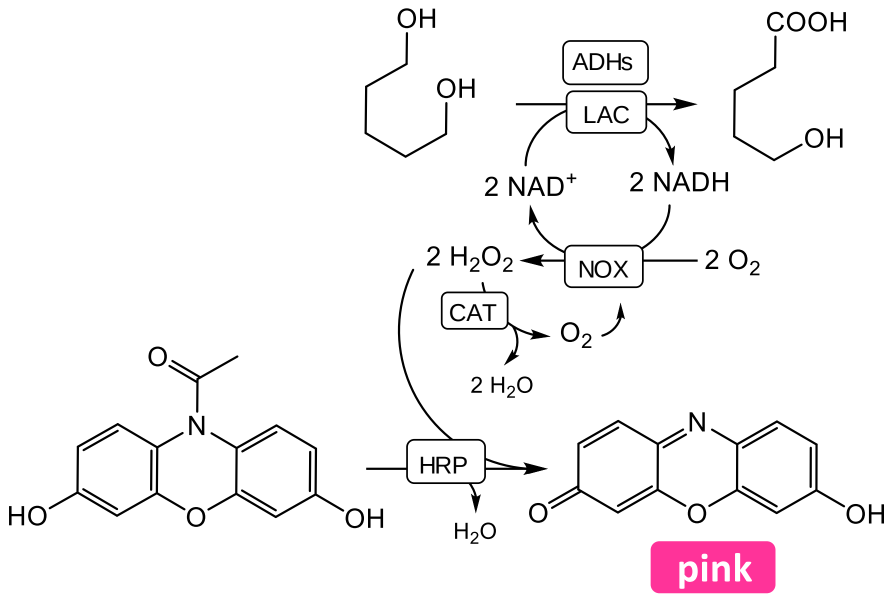

Figure S3. Colorimetric assay for measuring the hydrogen peroxide accumulation. Recording the absorbance increase at 560 nm due to the formation of resorufin. Reaction conditions consisted of 0.5 μg·mL^{-1} HRP, 20 mM 1,5-PD, 1 mM NAD^+, 0.15 mM FAD^+, and 50 μM Ampliflu™ Red in 100 mM sodium phosphate buffer pH 8.

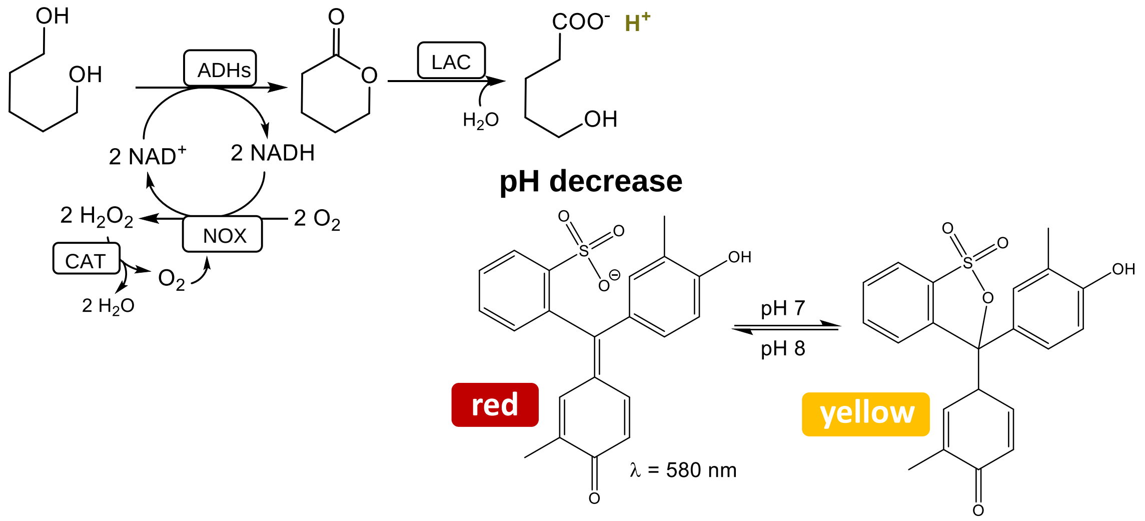

Figure S4. Colorimetric assay for measuring the 5-HP production. Recording the decrease in the absorbance at 580 nm and at the same time recording the absorbance at 340 nm to guarantee that there is no NADH accumulation since it also decreases the pH.

Reaction conditions consisted of 20 mM 1,5-PD, 1 mM NAD^+, 0.15 mM FAD^+, and 0.1 mM Cresol Red in sodium phosphate buffer 2.5 mM pH 8.

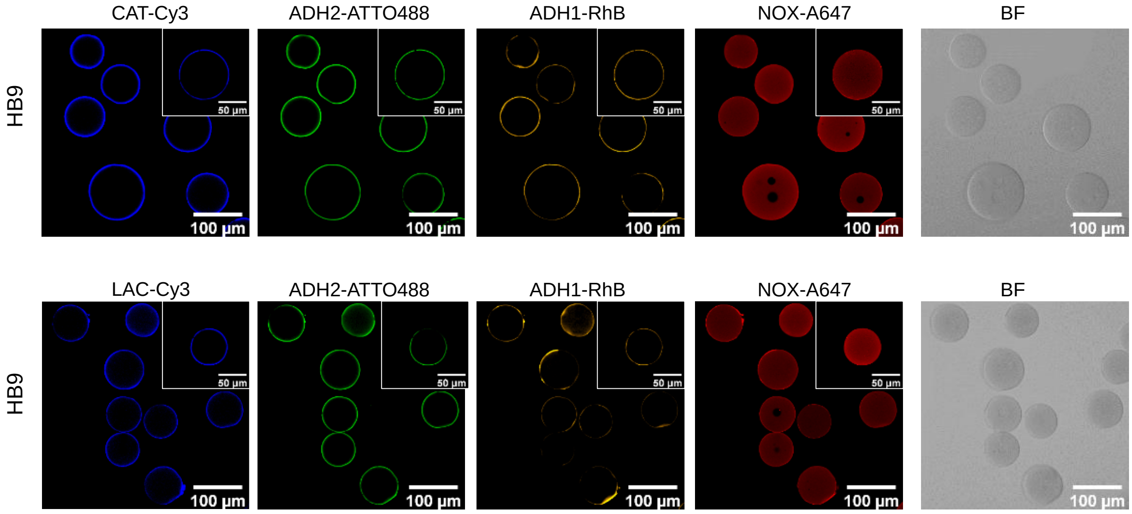

Figure S5. Uncontrolled spatial distribution of co-immobilized multi-enzyme system in HB9((ADH1-RhB), HB2 (ADH2-ATTO488), HB3 (NOX-A647), HB4 (LAC-Cy3) and HB5 (CAT-Cy3)).

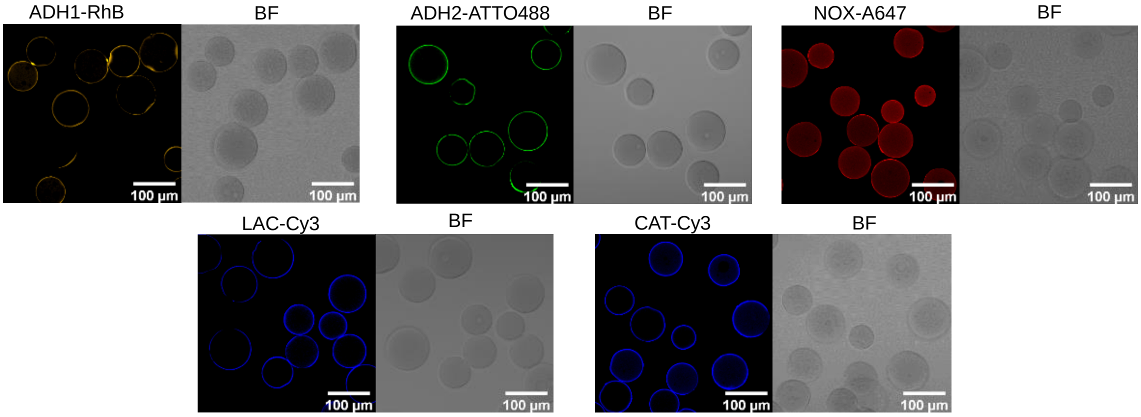

Figure S6. Spatial distribution of single immobilized enzymes (mixture of HB1 (ADH1-RhB), HB2 (ADH2-ATTO488), HB3 (NOX-A647), HB4 (LAC-Cy3) and HB5 (CAT-Cy3)).

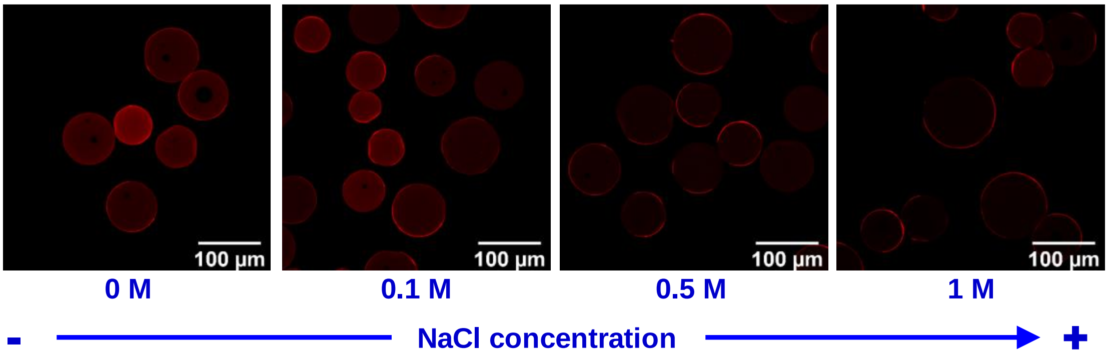

Figure S7. Control of the spatial distribution of NOX by ionic strength.

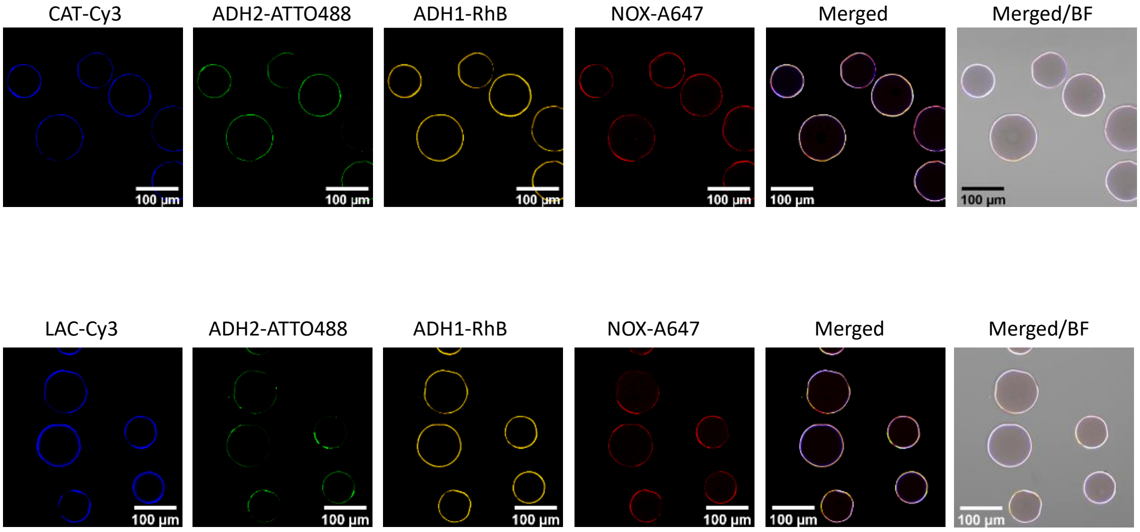

Figure S8. Controlled spatial distribution of co-immobilized multi-enzyme system in HB10 using 1M NaCl in the immobilization process.

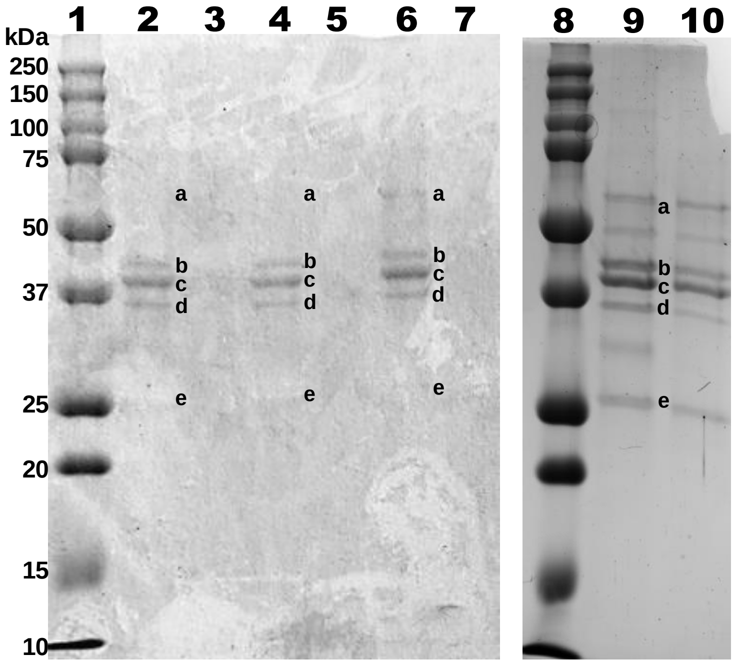

Figure S9. SDS-PAGE gels of HBs after five batch cycles. Lanes correspond to: 1, molecular weight marker; 2, Freshly prepared HB10; 3, HB10 after 5 batch reaction cycles; 4, Freshly prepared HB11; 5, HB11 after 5 batch reaction cycles; 6, Freshly prepared HB12; 7, HB12 after 5 batch reaction cycles; 8, molecular weight marker; 9, Freshly prepared HB13; 10, HB13 after 5 batch reaction cycles. a) CAT, 58 kDa, b) ADH2, 41 kDa, c) ADH1, 36 kDa, d) LAC, 35 kDa, and e) NOX, 23 kDa.

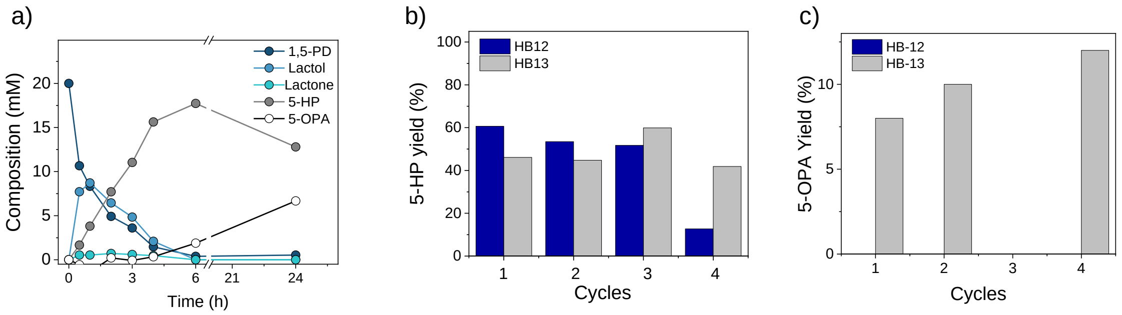

Figure S10. a Reaction-time course of HB13 at 20 mM of 1,5-PD, 1 mM NAD^+, 0.15 mM FAD^+ in 100 mM sodium phosphate buffer pH 8 at 30 ºC. **b**, Conversion yield of HB12 and HB13 after consecutive operational 4 h cycles at 10 mM 1,5-PD initial substrate concentration. **c**, 5-OPA yield of HB12 and HB13 after consecutive operational 4 h cycles at 10 mM 1,5-PD initial substrate concentration. Data in panels a,b,c represent the mean value and standard deviation (error bars) of two independent experiments.

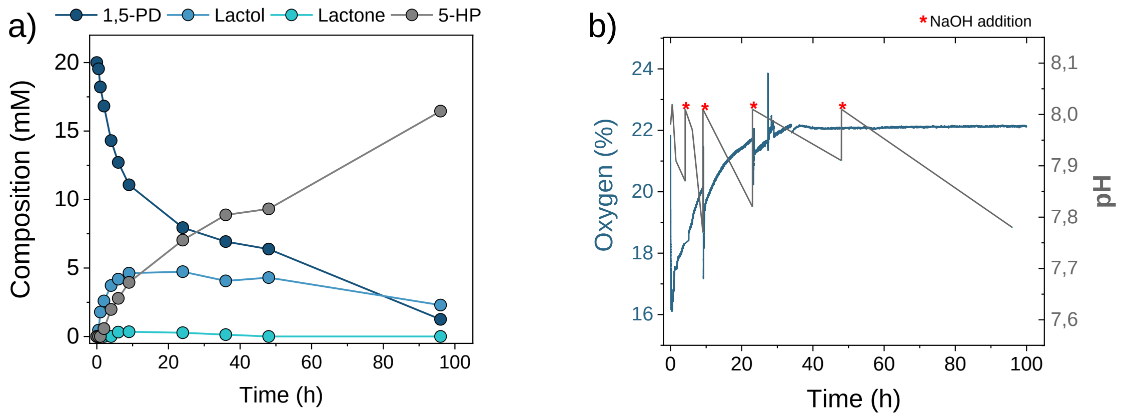

Figure S11. 30 mL scale batch reaction. **a**, Reaction time course, **b**, Oxygen and pH signal during the batch reaction. Reaction conditions consisted of 1.5 g of HB13 added to 30 mL of a reaction mixture composed of 20 mM 1,5-PD, 1 mM NAD^+, and 0.15 mM FAD^+ in 100 mM sodium phosphate buffer at pH 8 and 30 ºC. Data in panel a represents the mean value and standard deviation (error bars) of two independent experiments.

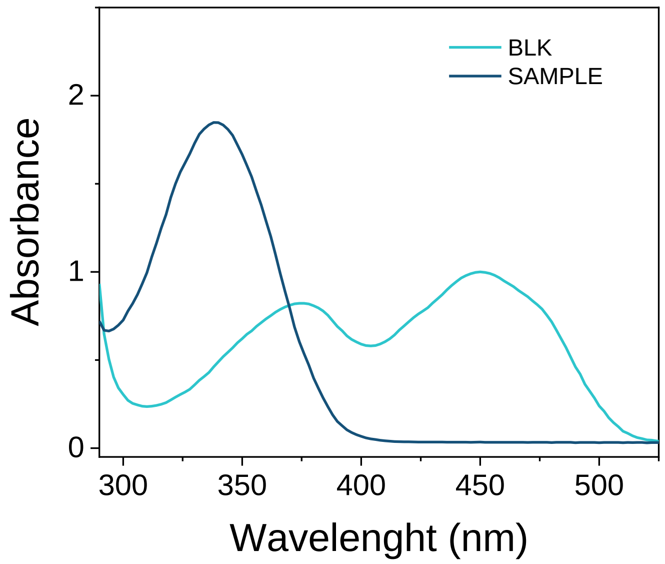

Figure S12. UV-Vis Spectra of the collected volume (sample) after the first test of the PBR flow reaction at 10 mM of substrate and 20 μL·min^{-1} flow rate. Consumed diol: 12.3%. As control (blank) we measured the reaction mixture before passing through the PBR.

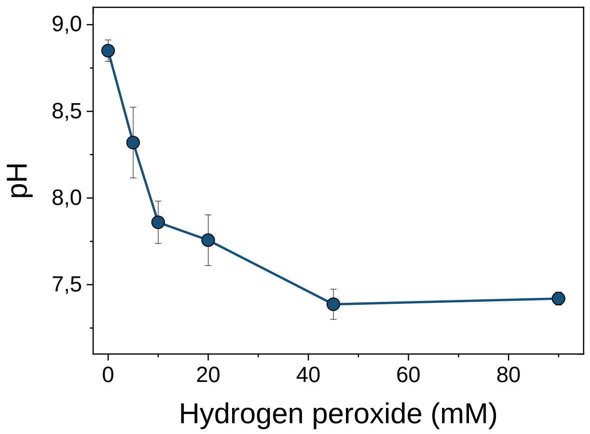

Figure S13. pH values of collected samples after PBR flow reaction at different hydrogen peroxide concentrations.

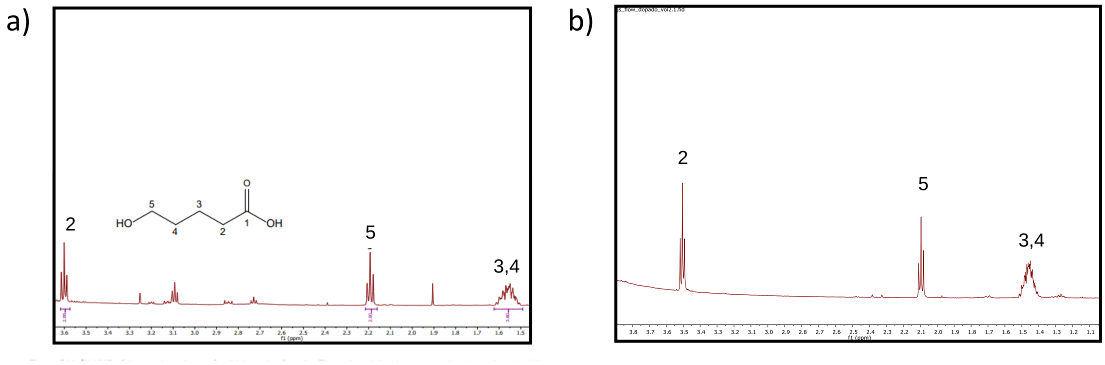

Figure S14. ^{1}H NMR of the reaction mixture of **a** soluble system reported in Velasco-Lozano et al.[1] **b**, PBR flow reaction. The assigned signals correspond to the produced 5-HP, 5-hydroxypentanoic acid. ^{1}H NMR (500 MHz, Deuterium Oxide) δ 3.60 (t, J = 6.3 Hz, 2H, H2), 2.19 (t, J = 7.1 Hz, 2H,H5), 1.56 (m, 4H,H3,H4).[2]

a)

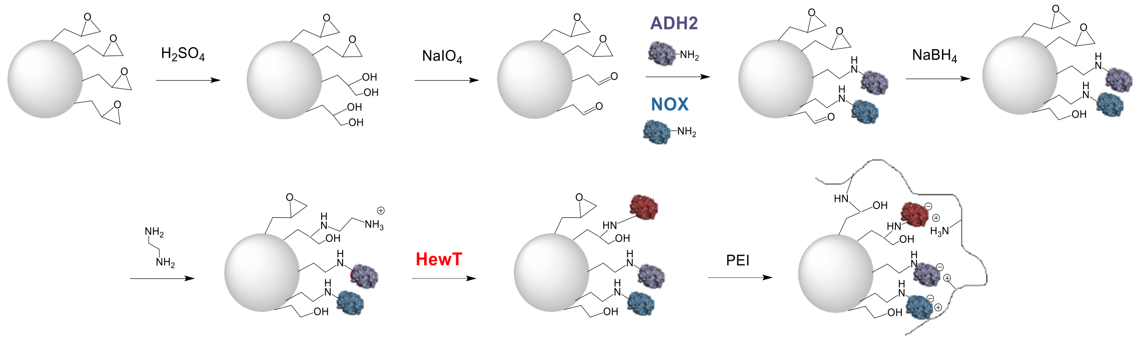

b)

Enzyme	Enzyme load (offered / final) (mg·g^{-1})	Ψ [a] (%)	Recovered activity [b] (U·g^{-1}) / (%)
ADH2	4 / 4[c]	>99	0.97 (65)
NOX	5 / 4.65[c]	93	1.5 (3.5)
HewT	5 / 3.85[c]	77	0.66 (19)

Figure S15. a Scheme of the immobilization protocol for the preparation of HB14. **b**, Immobilization parameters of HB14. [a] Immobilization yield, Ψ = (immobilized activity/offered activity) × 100. [b] (%) Recovered activity of the immobilized enzyme is defined as the coefficient between the specific activity of the immobilized enzymes and the specific activity of the soluble ones. [c] Total protein content.

Supplementary Tables

Table S1. JaCoP and Manders coefficients

Sample (M1-M2)	Pearson's coefficient	M1	M2	
NOX-ADH1	0,6	0,923	0,186	Manders' Coefficients (using threshold value of 11419 for imgNOX and 30642 for imgADH1)
NOX-CAT	0,578	0,805	0,216	Manders' Coefficients (using threshold value of 11419 for imgNOX and 21828 for imgCAT)
NOX-ADH2	0,647	0,732	0,359	Manders' Coefficients (using threshold value of 11419 for imgNOX and 6994 for imgADH2)
ADH1-ADH2	0,781	0,452	0,945	Manders' Coefficients (using threshold value of 30642 for imgADH1 and 6994 for imgADH2)
ADH1-CAT	0,857	0,755	0,889	Manders' Coefficients (using threshold value of 30642 for imgADH1 and 21828 for imgCAT)
ADH2-CAT	0,827	0,979	0,599	Manders' Coefficients (using threshold value of 6994 for imgADH2 and 21828 for imgCAT)
CAT-ADH2	0,752	0,558	0,978	Manders' Coefficients (using threshold value of 20328 for imgCAT and 7494 for imgADH2)
CAT-NOX	0,51	0,16	0,828	Manders' Coefficients (using threshold value of 20328 for imgCAT and 14538 for imgNOX)
CAT-ADH1	0,859	0,926	0,794	Manders' Coefficients (using threshold value of 20328 for imgCAT and 30336 for imgADH1)
ADH2-NOX	0,522	0,227	0,612	Manders' Coefficients (using threshold value of 7494 for imgADH2 and 14538 for imgNOX)
ADH2-ADH1	0,801	0,992	0,459	Manders' Coefficients (using threshold value of 7494 for imgADH2 and 30336 for imgADH1)
NOX-ADH1	0,571	0,989	0,169	Manders' Coefficients (using threshold value of 14538 for imgNOX and 30336 for imgADH1)
LAC-ADH2	0,615	0,551	0,661	Manders' Coefficients (using threshold value of 13052 for imgLAC and 5267 for imgADH2)
LAC-NOX	0,303	0,035	0,119	Manders' Coefficients (using threshold value of 13052 for imgLAC and 10524 for imgNOX)
LAC-ADH1	0,77	0,855	0,603	Manders' Coefficients (using threshold value of 13052 for imgLAC and 30311 for imgADH1)
ADH2-ADH1	0,74	0,941	0,531	Manders' Coefficients (using threshold value of 5267 for imgADH2 and 30311 for imgADH1)
ADH2-NOX	0,499	0,203	0,532	Manders' Coefficients (using threshold value of 5267 for imgADH2 and 10524 for imgNOX)
ADH1-NOX	0,526	0,128	0,82	Manders' Coefficients (using threshold value of 30311 for imgADH1 and 10524 for imgNOX)
LAC-ADH2	0,569	0,221	0,965	Manders' Coefficients (using threshold value of 12470 for imgLAC and 7340 for imgADH2)
LAC-NOX	0,539	0,27	0,677	Manders' Coefficients (using threshold value of 12470 for imgLAC and 7052 for imgNOX)
LAC-ADH1	0,776	0,754	0,704	Manders' Coefficients (using threshold value of 12470 for imgLAC and 31887 for imgADH1)
ADH2-NOX	0,543	0,706	0,37	Manders' Coefficients (using threshold value of 7340 for imgADH2 and 7052 for imgNOX)
ADH2-ADH1	0,62	0,987	0,188	Manders' Coefficients (using threshold value of 7340 for imgADH2 and 31887 for imgADH1)
NOX-ADH1	0,685	0,964	0,344	Manders' Coefficients (using threshold value of 7052 for imgNOX and 31887 for imgADH1)

Table S1: Manders' and Person coefficients measure the degree of co-localization of objects in confocal dual-color images. Manders' coefficient means the fraction of co-localizing objects in each component of the dual-channel image.[3] For example the fraction of pixels where the fluorescence of one enzyme (M1) is detected together with the fluorescence of the other enzyme (M2). Person provides a linear association between two variables, in this case, the fluorescence of one labeled enzyme regarding the other labeled one. [3]

Table S2. Reaction operation conditions are considered for green metric parameters.

Condition	Soluble[1]	Batch	Flow
Substrate concentration 1,5-PD (mM)	20	10	20
Mass of enzymes (µg)	0.23	0.62	0.93
Reactor bead volume (mL)	0.5	0.3	1.5
Total processed volume (mL)	0.5	1.2	14.4
Operation time (h)	24	24	24
Substrate feeding rate (µL/min)	Not applicable	Not applicable	10
Reaction cycles	1	4	Not applicable
H_2O_2 (mM)	0	0	45
Others	1 mM NAD^+, and 0.15 mM FAD^+ in 100 mM sodium phosphate buffer at pH 8 and 30 ºC		

[1] These results are found in our previous publication.[1]

Table S3. Operational stability of ADH2, NOX, and HewT after the telescoped flow reaction for the synthesis of 5-AP. [a] Retained activity (%) refers to the enzyme activity detected on the HB after 72 h at 30 ºC in the flow reactor.

Enzyme	PBR	Activity before flow reaction ($U \cdot g^{-1}$)	Activity after flow reaction ($U \cdot g^{-1}$)	[a] Retained activity (%)
ADH2	PBR1 (HB13)	0.86	0.28	33
NOX		0.68	0.51	75
ADH2	PBR2 (HB14)	0.97	0.13	14
NOX		1.5	0.84	56
HewT		0.66	0.42	64

All data herein presented correspond to the mean value of three independent enzyme activity and protein concentration assays. In all cases, the standard deviation was never higher than 5% of the mean value.

Supplementary References

(1) Velasco-Lozano, S.; Santiago-Arcos, J.; Grazia Rubanu, M.; López-Gallego, F., Cell-Free Biosynthesis of ω-Hydroxy Acids Boosted by a Synergistic Combination of Alcohol Dehydrogenases. *ChemSusChem* **2022,** *15* (9), e202200397.

(2) Zhou, Z.-z.; Liu, M.; Lv, L.; Li, C.-J., Silver(I)-Catalyzed Widely Applicable Aerobic 1,2-Diol Oxidative Cleavage. *Angew. Chem. Int. Ed.* **2018,** *57* (10), 2616-2620.

(3) Dunn, K. W.; Kamocka, M. M.; McDonald, J. H., A practical guide to evaluating colocalization in biological microscopy. *Am. J. Physiol. Cell Physiol.* **2011,** *300* (4), C723-42.
